# Supplementary material for: Hidden diversity and potential ecological function of phosphorus acquisition genes in widespread terrestrial bacteriophages
Source: Nat Commun. 2024 Apr 2;15:2827. doi: 10.1038/s41467-024-47214-7 (PMC10987575; doi:10.1038/s41467-024-47214-7)
Supplement: Supplementary file 1 — Supplementary Information [file 41467_2024_47214_MOESM1_ESM.pdf]

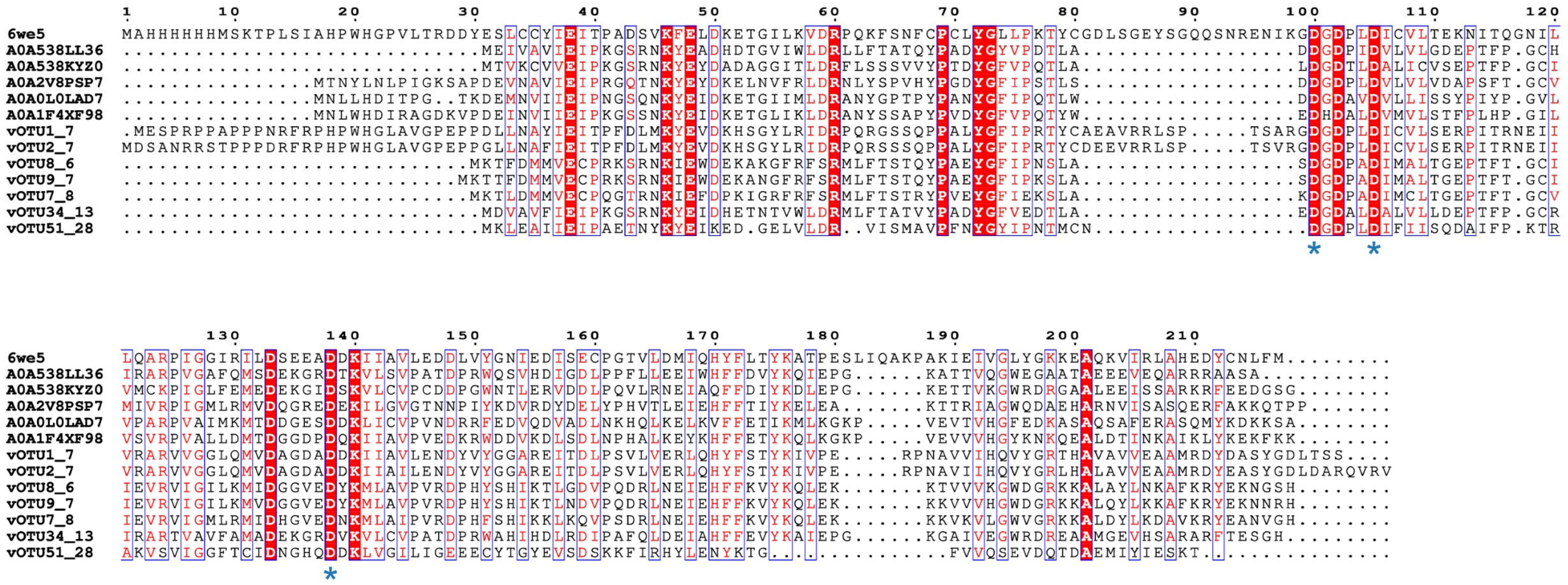

Supplementary Fig. 1 | Family I PPa protein alignment and conserved residues identified in prokaryotic and phage sequences. Positions of conserved active residues coordinating Mg^{2+}/Mn^{2+} (identified in the soluble Family I PPa)[1] are marked with blue asterisks below the alignment. Residues of the same type are highlighted in red background and highly conserved residues are shown in red. Those sequences whose names begin with vOTU are phage sequences from this study, while the others are prokaryotic reference sequences from the UniProt database. PPa, inorganic pyrophosphatase.

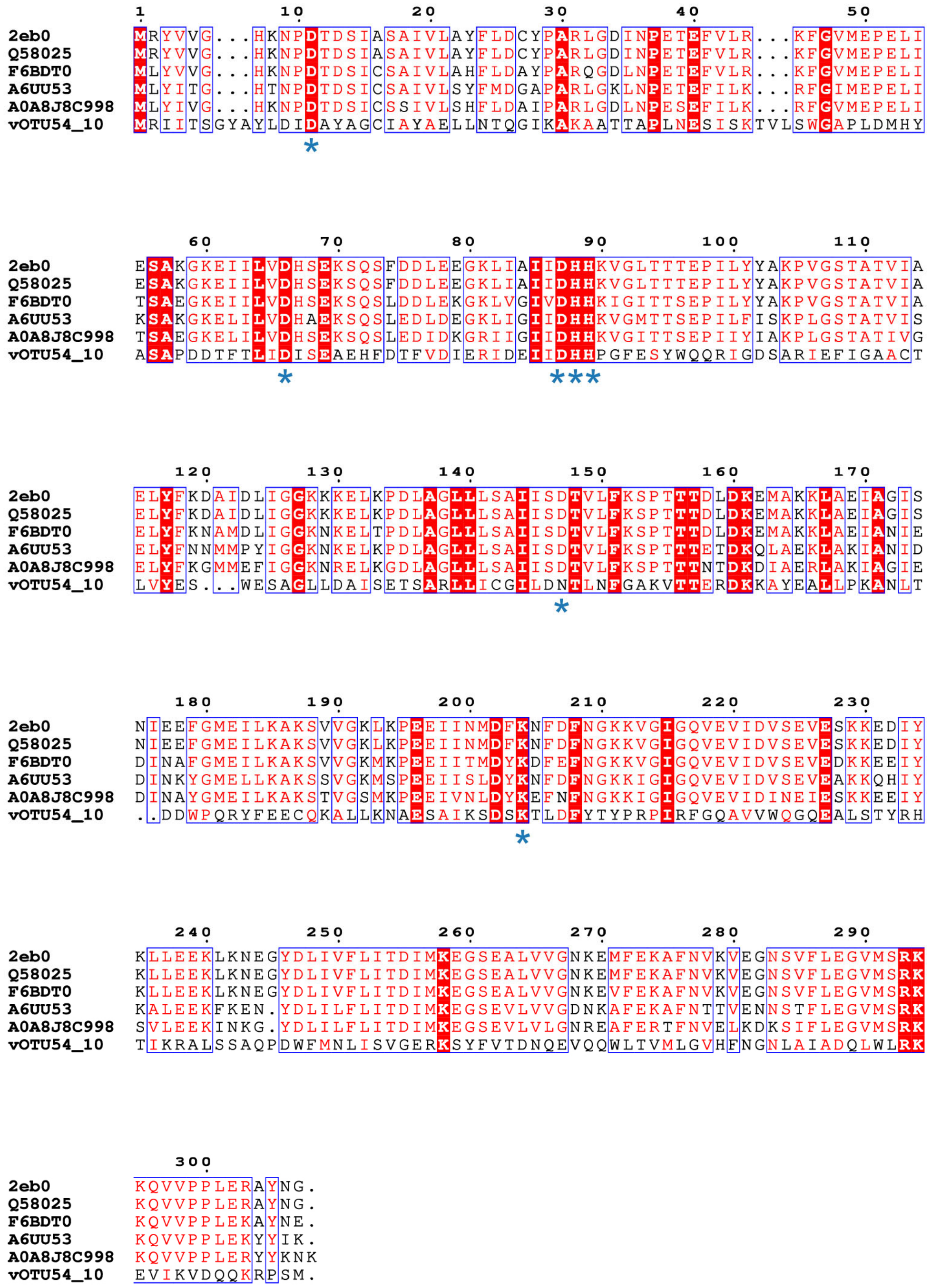

Supplementary Fig. 2 | Family II PPa protein alignment and conserved residues identified in prokaryotic and phage sequences. Positions of conserved active residues (identified in the soluble Family II PPa)[1] are marked with blue asterisks below the alignment. Residues of the same type are highlighted in red background and highly conserved residues are shown in red.

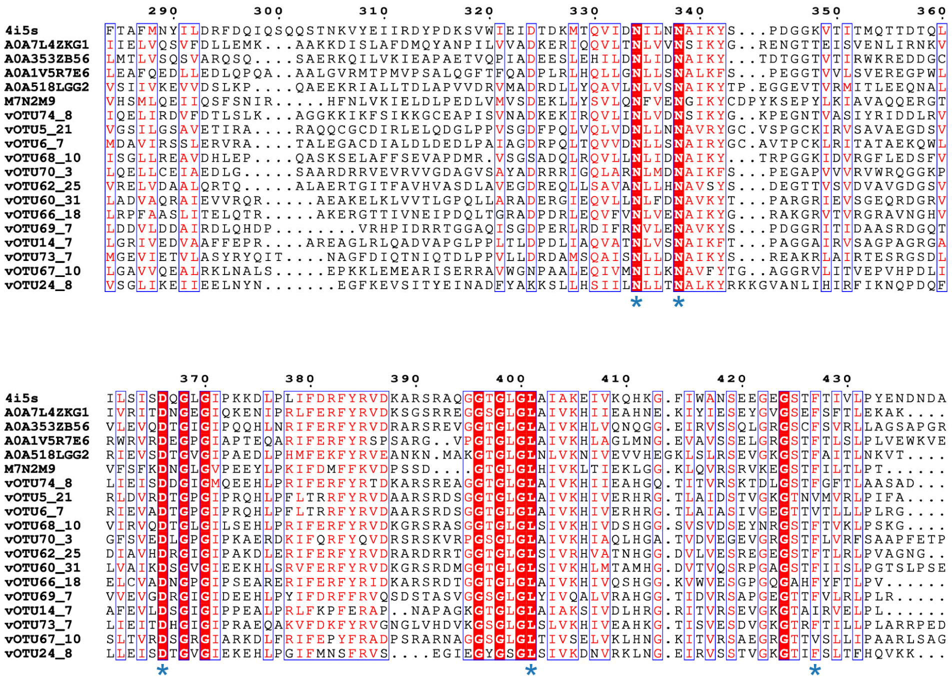

Supplementary Fig. 3 | PhoR protein alignment and conserved residues identified in prokaryotic and phage sequences. Positions of conserved ATP-binding residues[2] are marked with blue asterisks below the alignment. Residues of the same type are highlighted in red background and highly conserved residues are shown in red. PhoR, phosphate regulon sensor histidine kinase.

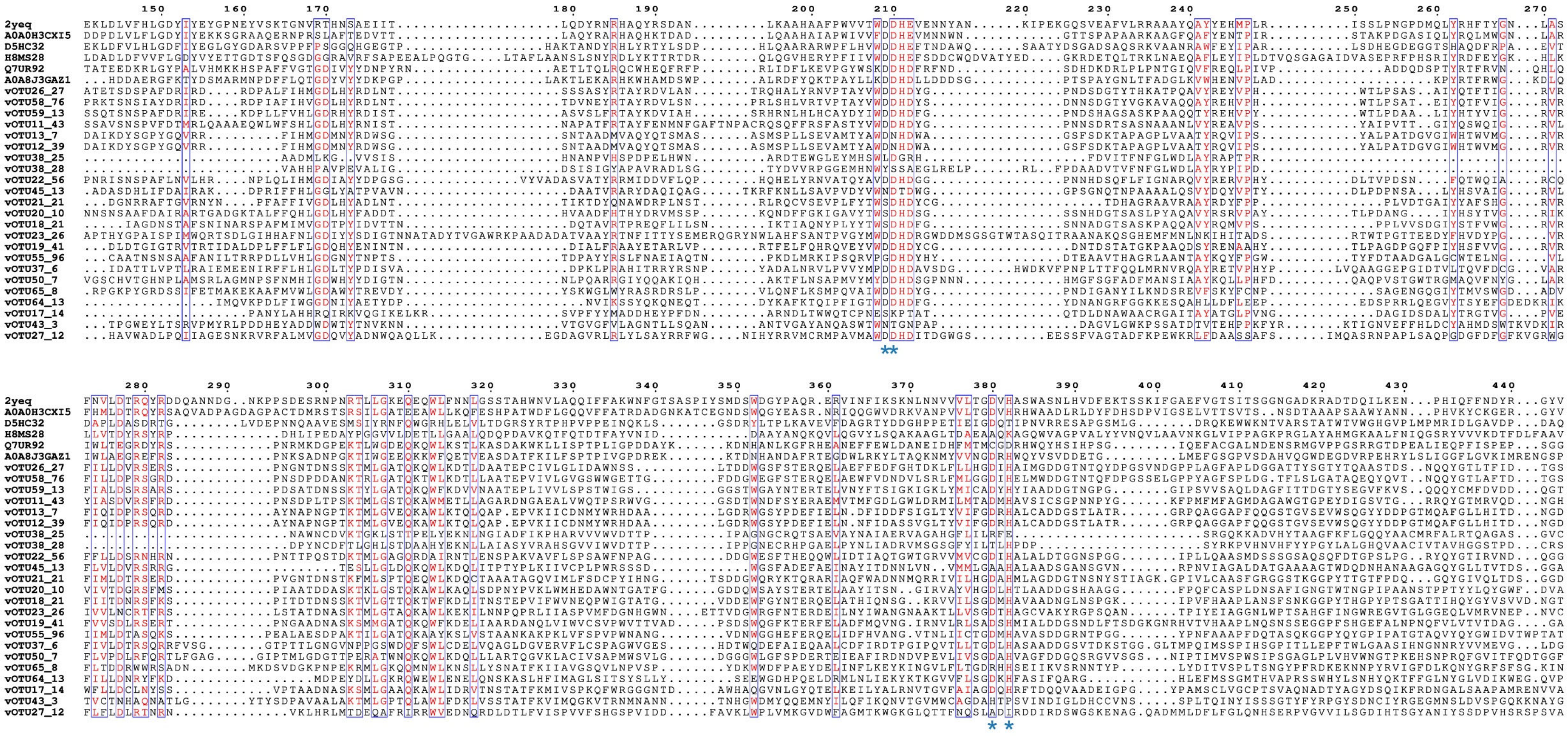

Supplementary Fig. 4 | PhoD protein alignment and conserved residues identified in prokaryotic and phage sequences. Positions of active residues[3] coordinating Fe^{3+} and Ca^{2+} are marked with blue asterisks below the alignment. Highly conserved residues are shown in red. PhoD, alkaline phosphatase.

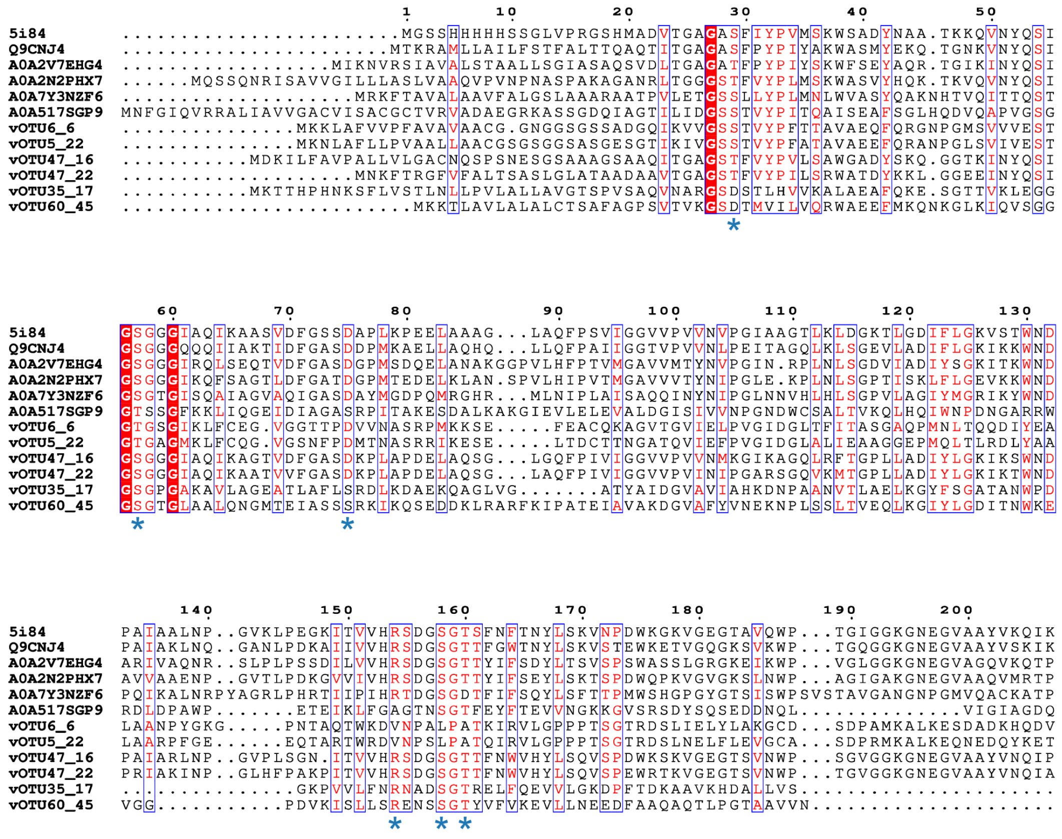

Supplementary Fig. 5 | PstS protein alignment and conserved residues identified in prokaryotic and phage sequences. Positions of conserved active residues involving in phosphate binding[4] are marked with blue asterisks below the alignment. Residues of the same type are highlighted in red background and highly conserved residues are shown in red. PstS, phosphate-specific transport system subunit S.

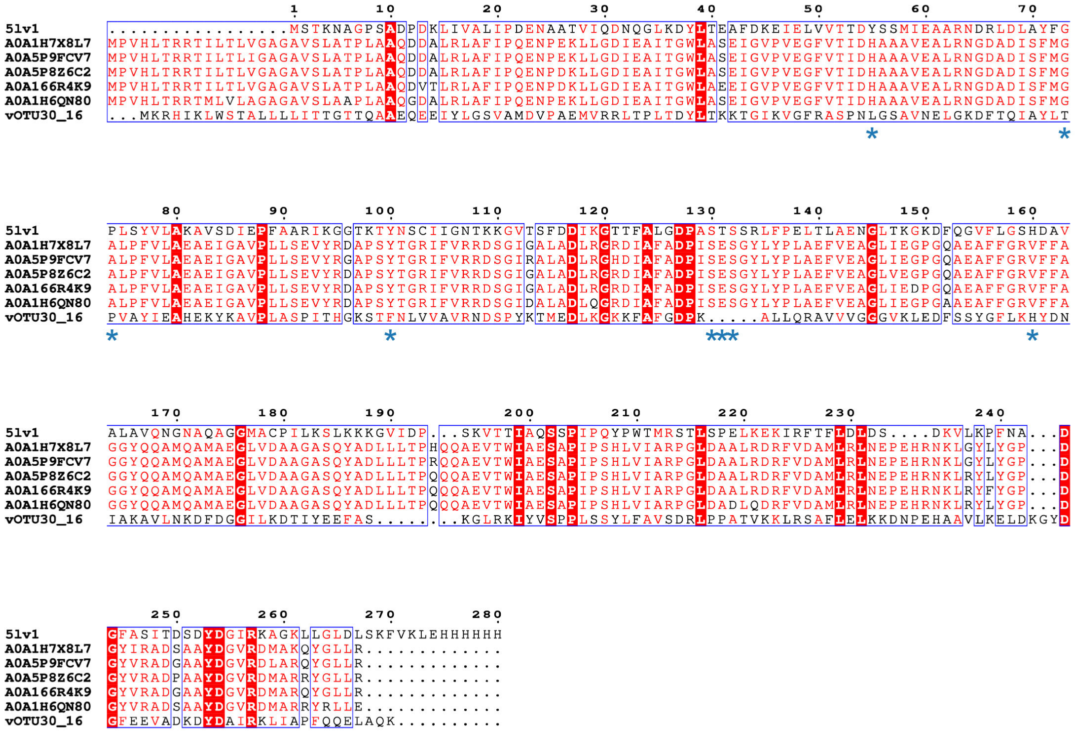

Supplementary Fig. 6 | PhnD protein alignment and conserved residues identified in prokaryotic and phage sequences. Positions of conserved active residues that form H-bonds with ligand oxygen atoms[5] are marked with blue asterisks below the alignment. Residues of the same type are highlighted in red background and highly conserved residues are shown in red. PhnD, phosphonate transporter subunit D.

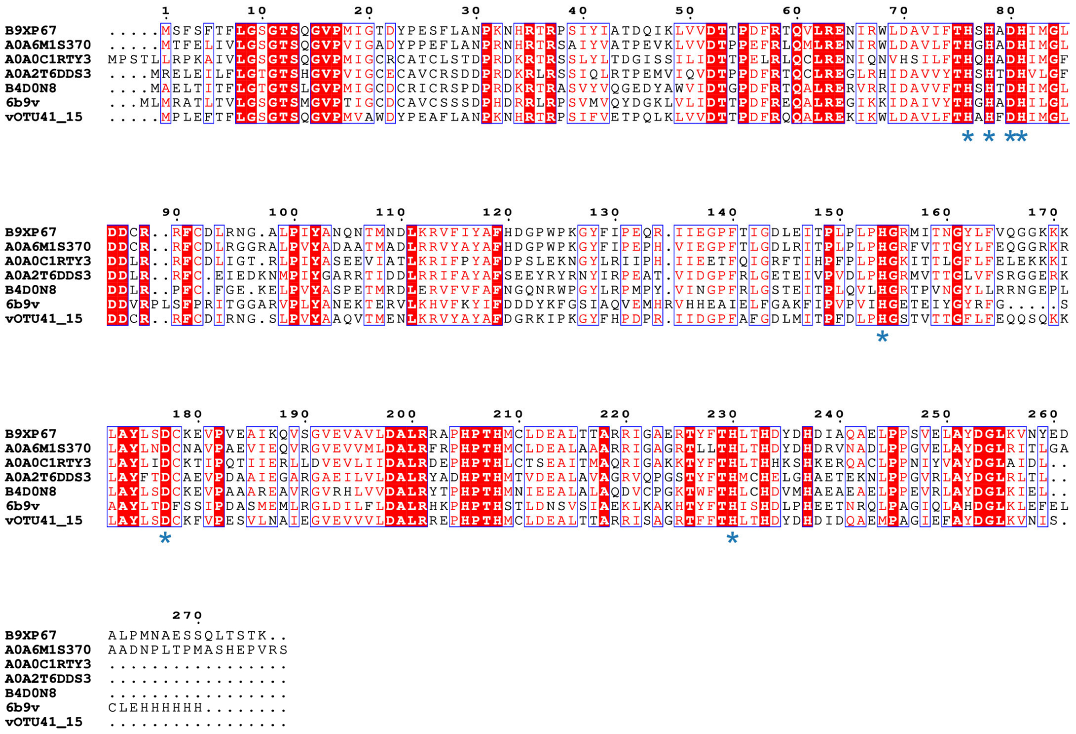

Supplementary Fig. 7 | PhnP protein alignment and identified conserved residues in prokaryotic and phage sequences. Positions of conserved active residues involved in Mg^{2+} coordination[6] are marked with blue asterisks below the alignment. Residues of the same type are highlighted in red background and highly conserved residues are shown in red. PhnP, C-P lyase subunit P.

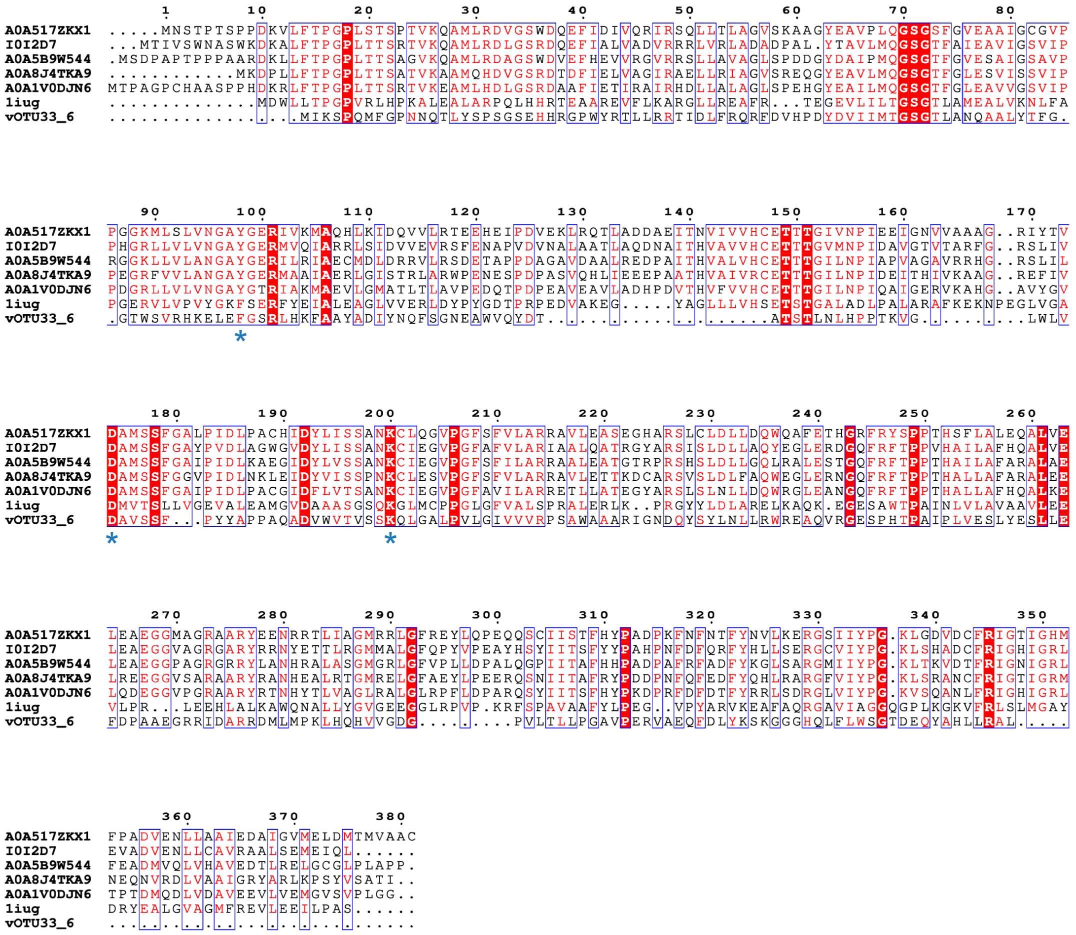

Supplementary Fig. 8 | PhnW protein alignment and conserved residues identified in prokaryotic and phage sequences. Positions of conserved active residues detected from the Catalytic Site Atlas (CSA) database integrated in Phyre2[7] are marked with blue asterisks below the alignment. Residues of the same type are highlighted in red background and highly conserved residues are shown in red. PhnW, 2-aminoethylphosphonate-pyruvate transaminase.

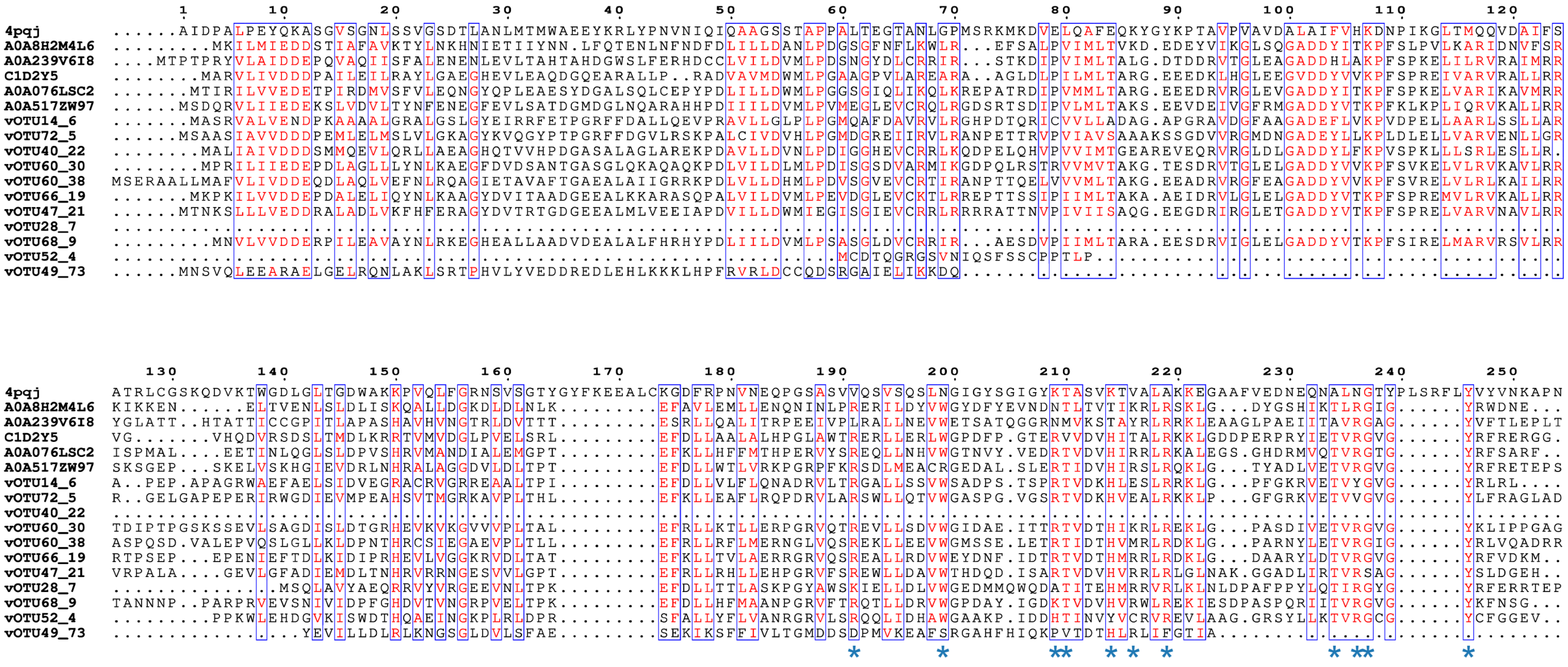

Supplementary Fig. 9 | PhoB protein alignment and conserved residues identified in prokaryotic and phage sequences. Positions of residues interacting with the phosphate or ribose moiety[8] are marked with blue asterisks below the alignment. Highly conserved residues are shown in red. PhoB, phosphate regulon response regulator.

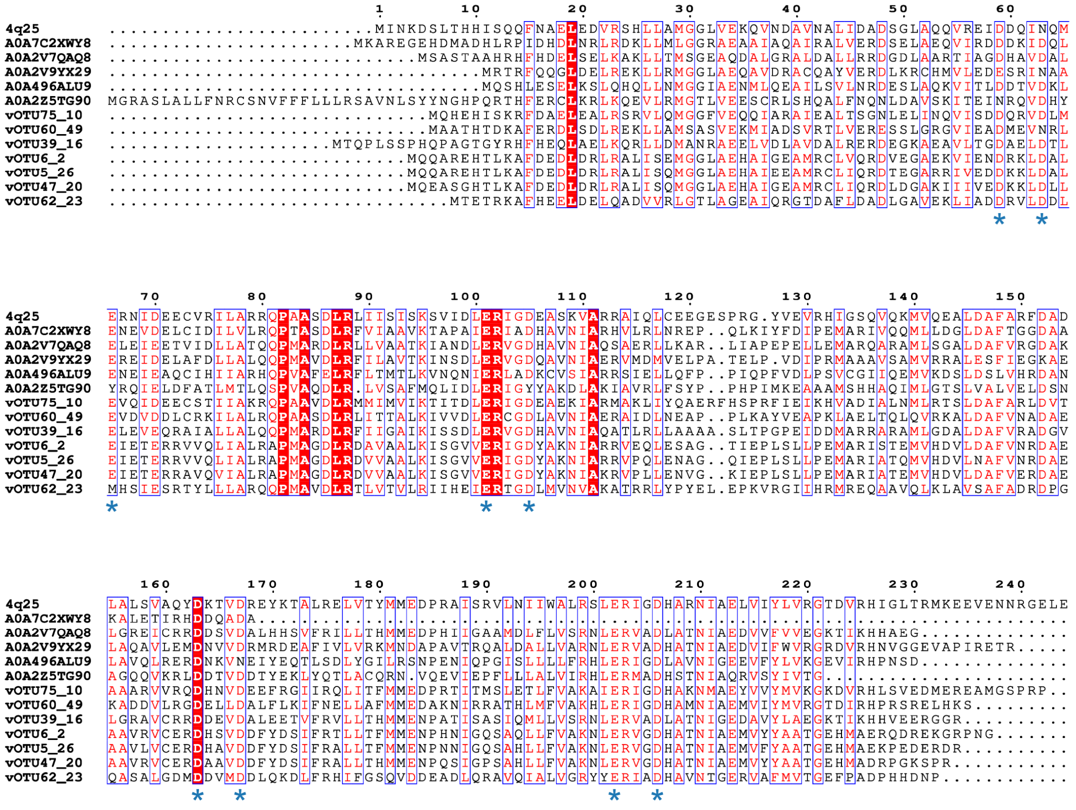

Supplementary Fig. 10 | PhoU protein alignment and conserved residues identified in prokaryotic and phage sequences. Positions of residues that form the metal cluster binding sites[9] are marked with blue asterisks below the alignment. Residues of the same type are highlighted in red background and highly conserved residues are shown in red. PhoU, PhoR/PhoB inhibitor protein.

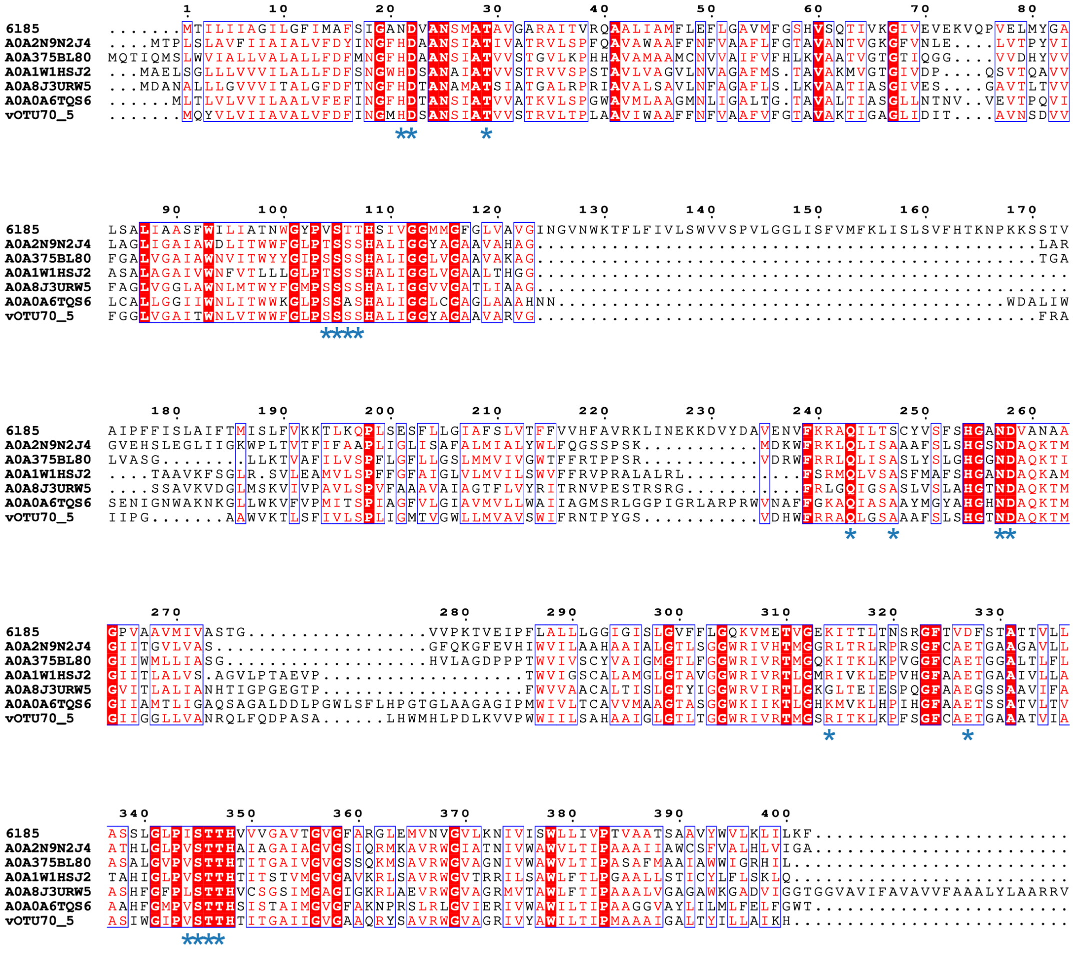

Supplementary Fig. 11 | PiT protein alignment and conserved residues identified in prokaryotic and phage sequences. The residues involved in phosphate and Na^+ coordination[10] are marked with blue asterisks below the alignment. Residues of the same type are highlighted in red background and highly conserved residues are shown in red. PiT, phosphate inorganic transporter.

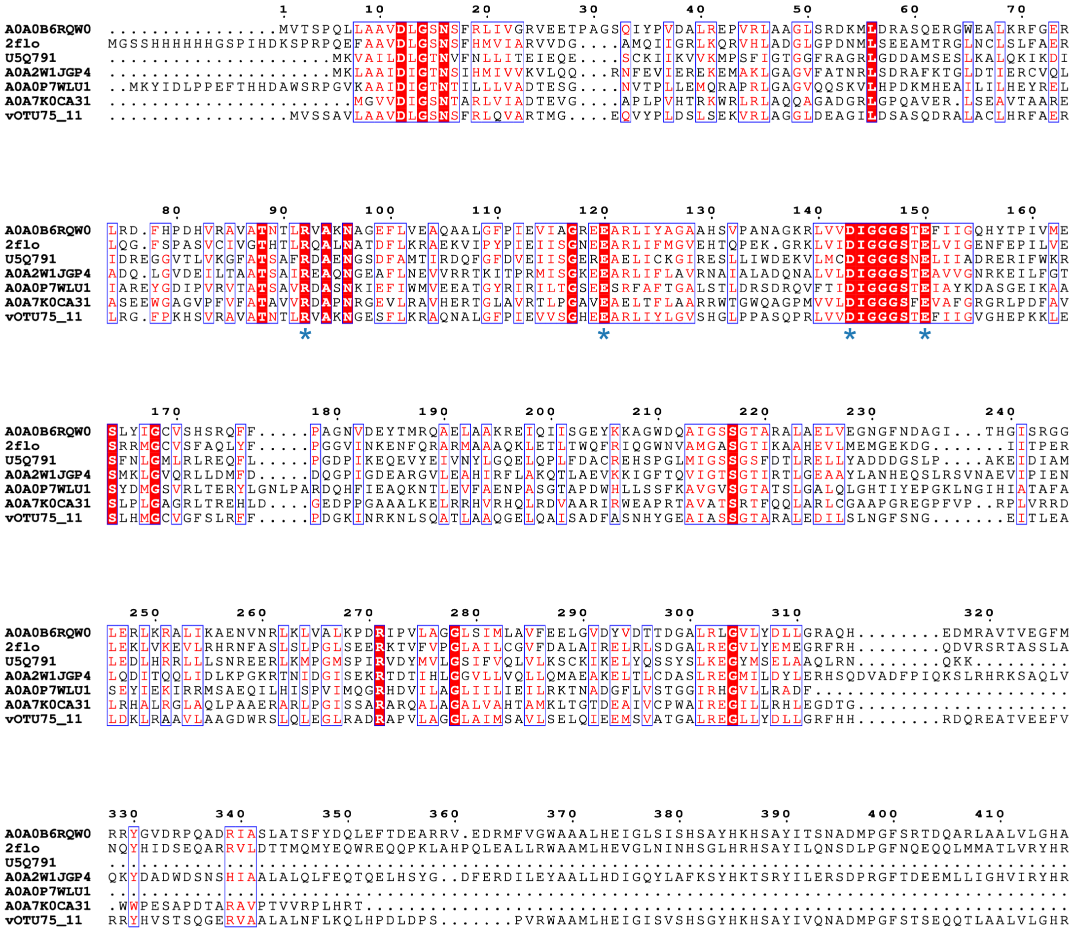

Supplementary Fig. 12 | Ppx protein alignment and conserved residues identified in prokaryotic and phage sequences. The potential Mg^{2+}-coordinating residues and putative catalytic sites[11] are marked with blue asterisks below the alignment. Residues of the same type are highlighted in red background and highly conserved residues are shown in red. Ppx, exopolyphosphatase.

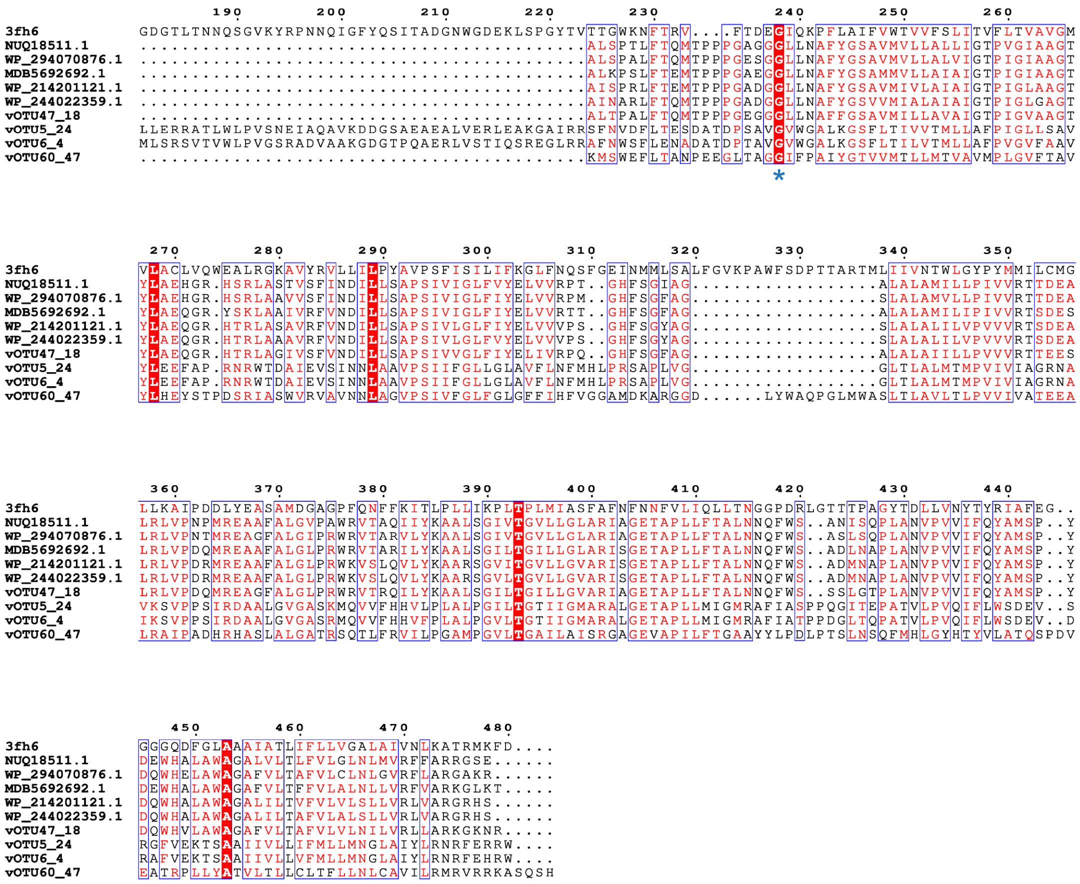

Supplementary Fig. 13 | PstA protein alignment and conserved residues identified in prokaryotic and phage sequences. Positions of conserved active residues detected from the CSA database integrated in Phyre2[7] are marked with blue asterisks below the alignment. Residues of the same type are highlighted in red background and highly conserved residues are shown in red. PstA, phosphate-specific transport system subunit A.

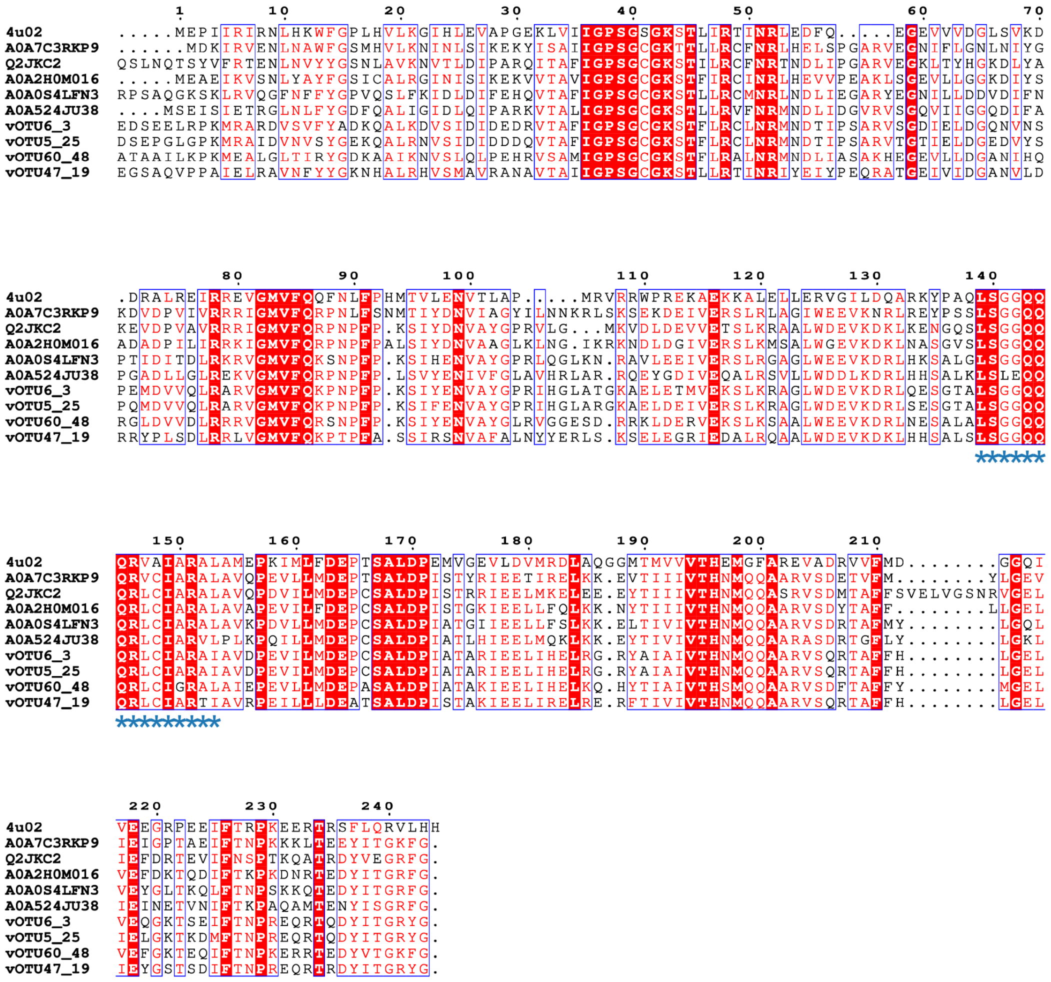

Supplementary Fig. 14 | PstB protein alignment and conserved residues identified in prokaryotic and phage sequences. Positions of conserved active residues detected from the CSA database integrated in Phyre2[7] are marked with blue asterisks below the alignment. Residues of the same type are highlighted in red background and highly conserved residues are shown in red. PstB, phosphate-specific transport system subunit B.

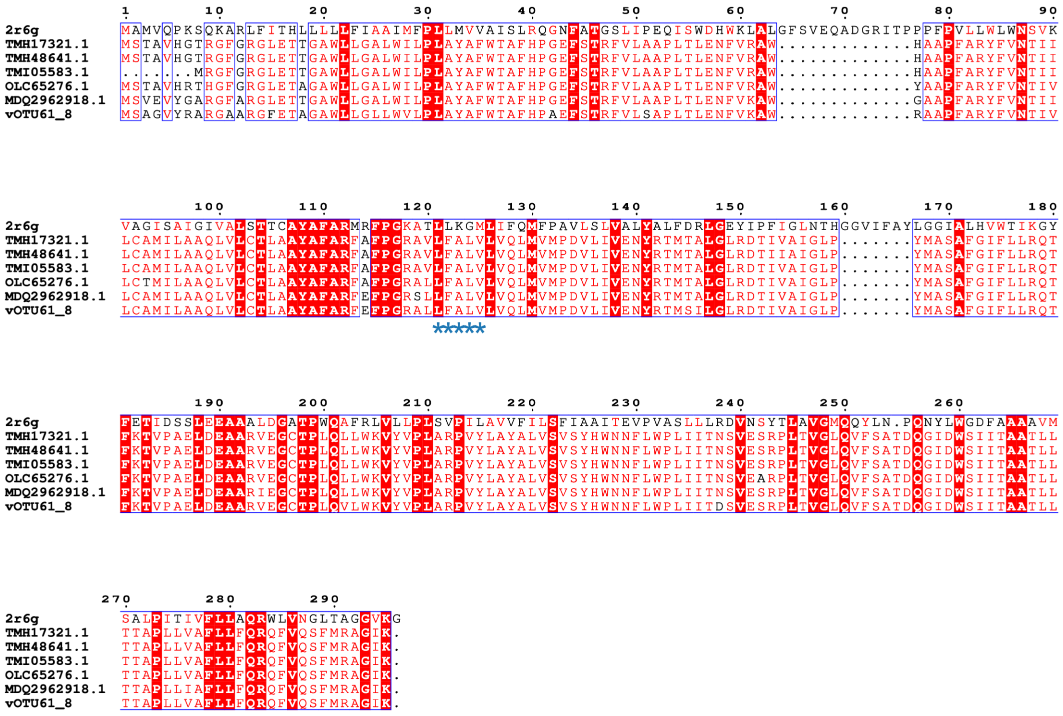

Supplementary Fig. 15 | UgpE protein alignment and conserved residues identified in prokaryotic and phage sequences. Positions of conserved active residues detected from the CSA database integrated in Phyre2[7] are marked with blue asterisks below the alignment. Residues of the same type are highlighted in red background and highly conserved residues are shown in red. UgpE, glycerol-3-phosphate transporter subunit E.

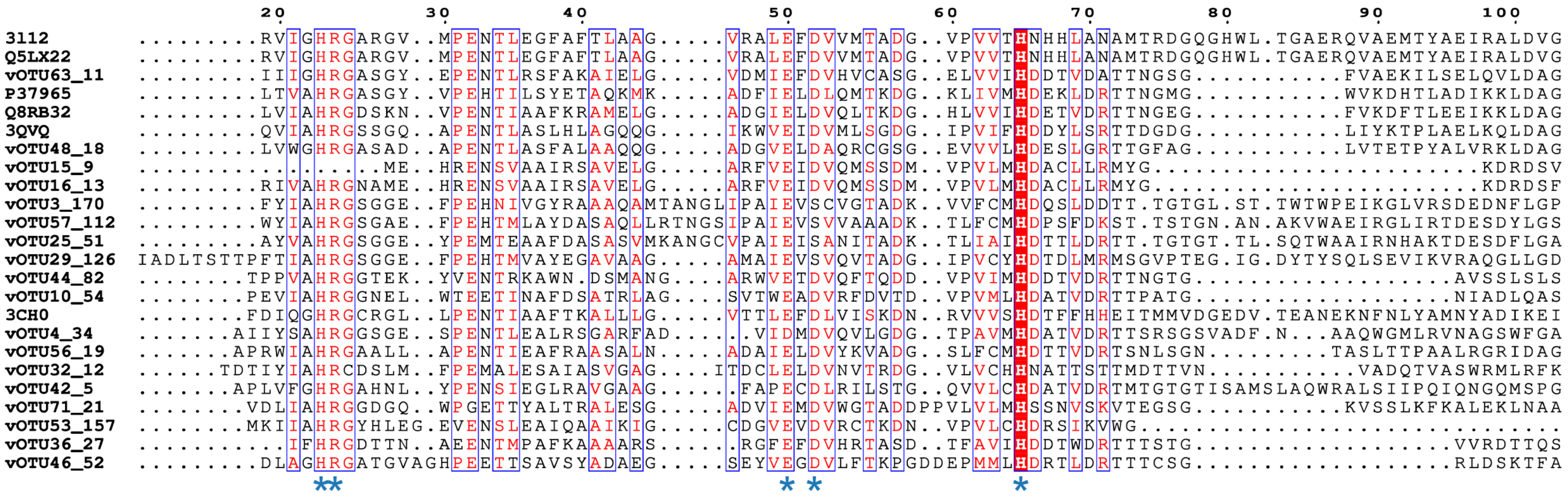

Supplementary Fig. 16 | UgpQ protein alignment and conserved residues identified in prokaryotic and phage sequences. Positions of conserved active residues verified by site-directed mutagenesis[12] are marked with blue asterisks below the alignment. Residues of the same type are highlighted in red background and highly conserved residues are shown in red. UgpQ, glycerophosphoryl diester.

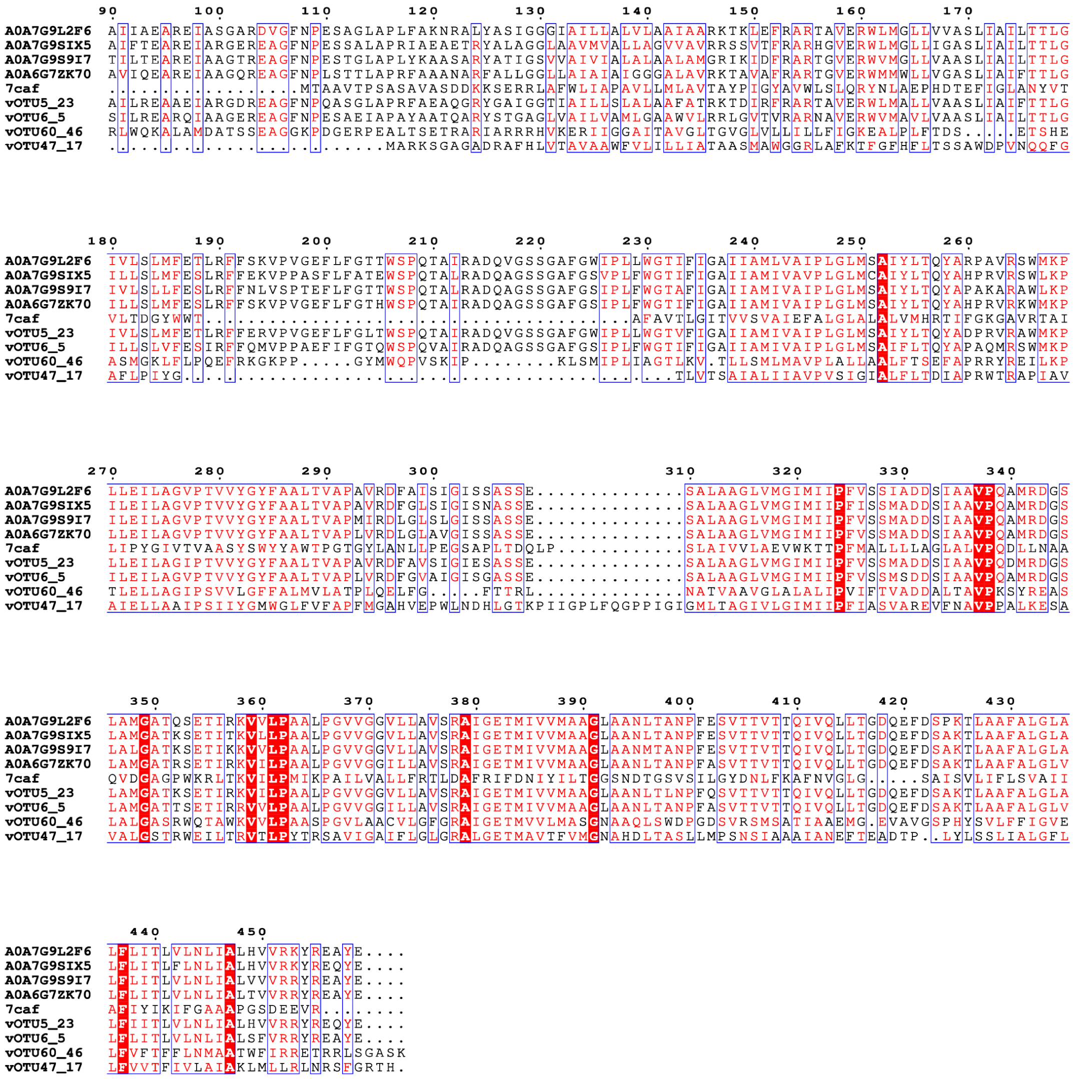

Supplementary Fig. 17 | PstC protein alignment and conserved residues identified in prokaryotic and phage sequences. Residues of the same type are highlighted in red background and highly conserved residues are shown in red. PstC, phosphate-specific transport system subunit C.

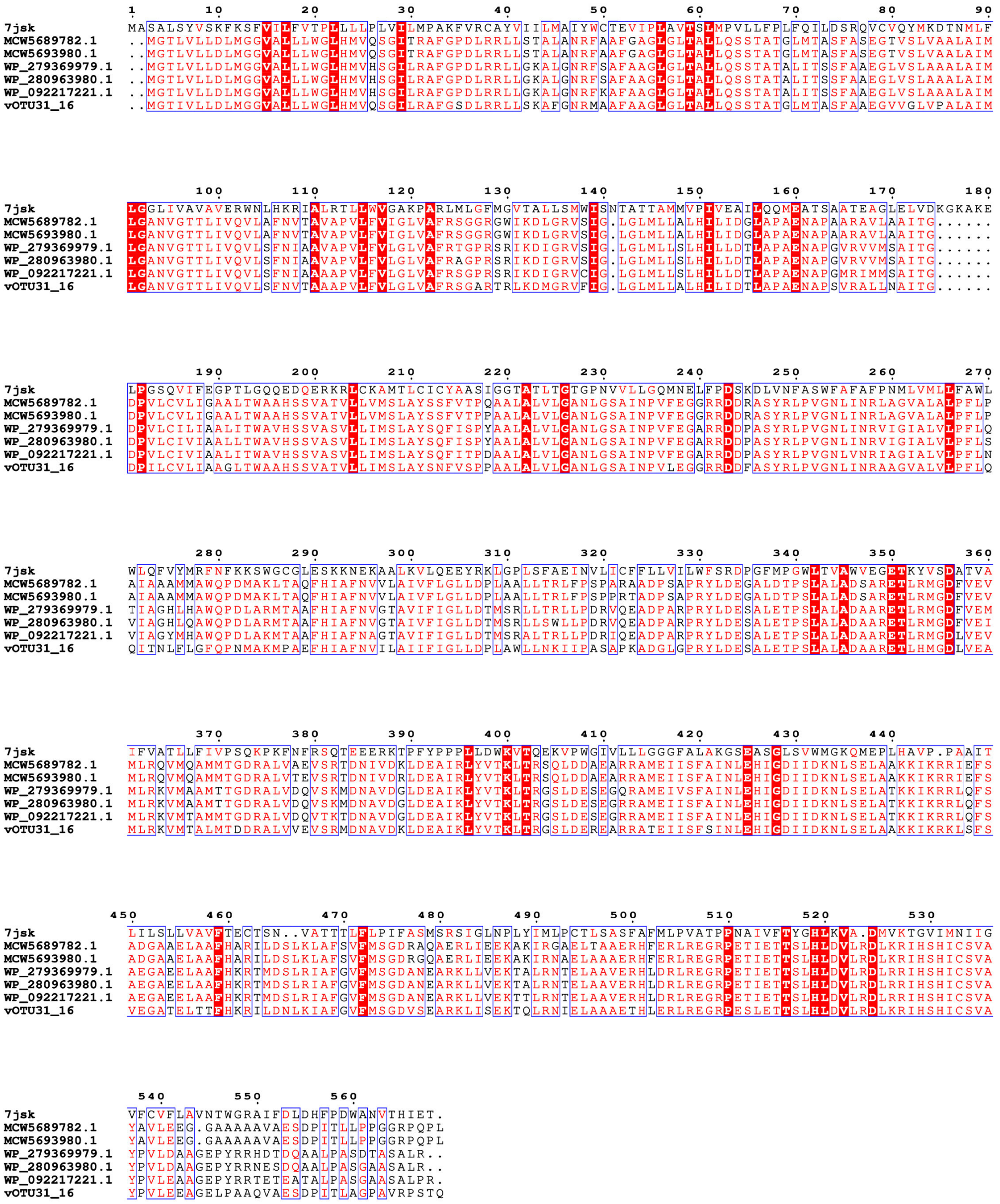

Supplementary Fig. 18 | YjbB protein alignment and conserved residues identified in prokaryotic and phage sequences. Residues of the same type are highlighted in red background and highly conserved residues are shown in red. YjbB, phosphate:Na+ symporter.

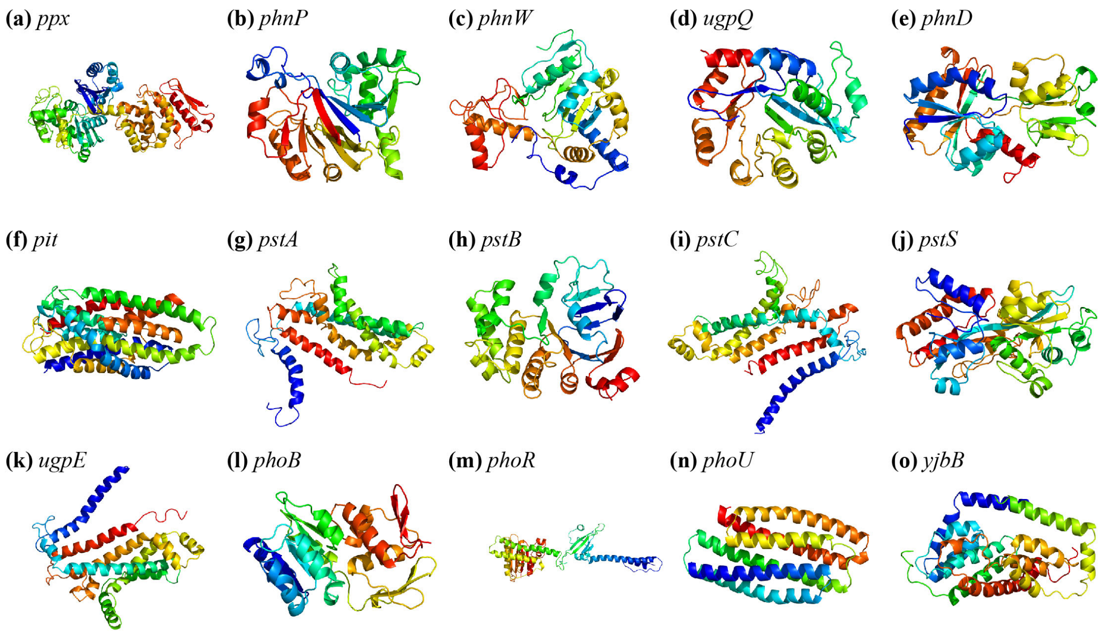

Supplementary Fig. 19 | Computational protein models of 15 kinds of P-acquisition AMGs. Helices and sheets are colored in a rainbow scheme (from the N terminus in red to the C terminus in blue). Detailed information of individual proteins is provided in Supplementary Data 12. *ppx*, exopolyphosphatase; *phnP*, C-P lyase subunit P; *phnW*, 2-aminoethylphosphonate-pyruvate transaminase; *ugpQ*, glycerophosphoryl diester; *phnD*, phosphonate transporter subunit D; *pit*, phosphate inorganic transporter; *pstA*, phosphate-specific transport system subunit A; *pstB*, phosphate-specific transport system subunit B; *pstC*, phosphate-specific transport system subunit C; *pstS*, phosphate-specific transport system subunit S; *ugpE*, glycerol-3-phosphate transporter subunit E; *phoB*, phosphate regulon response regulator; *phoR*, phosphate regulon sensor histidine kinase; *phoU*, PhoR/PhoB inhibitor protein; *yjbB*, phosphate:Na^+ symporter.

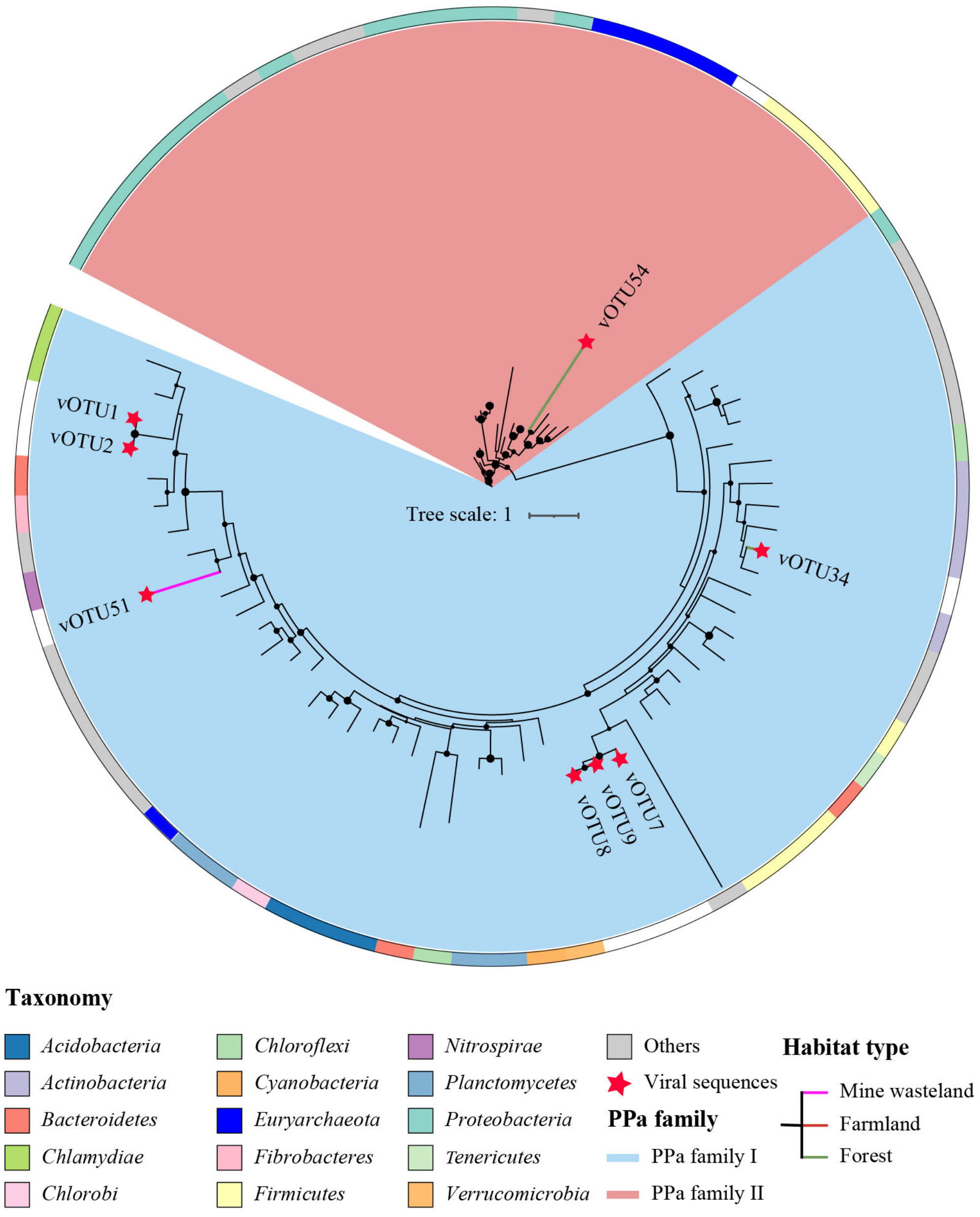

Supplementary Fig. 20 | Phylogenetic tree of PPa. Bootstrap values > 75% are labeled on the nodes. PPa encoded by the P-acquisition vOTUs identified in this study are labeled with stars and their habitat origins are indicated by colored branches. Branches colored in black represent the phylogenetic placement of the reference prokaryotic sequences. Outside rings denote the taxonomic affiliations of the prokaryotic proteins.

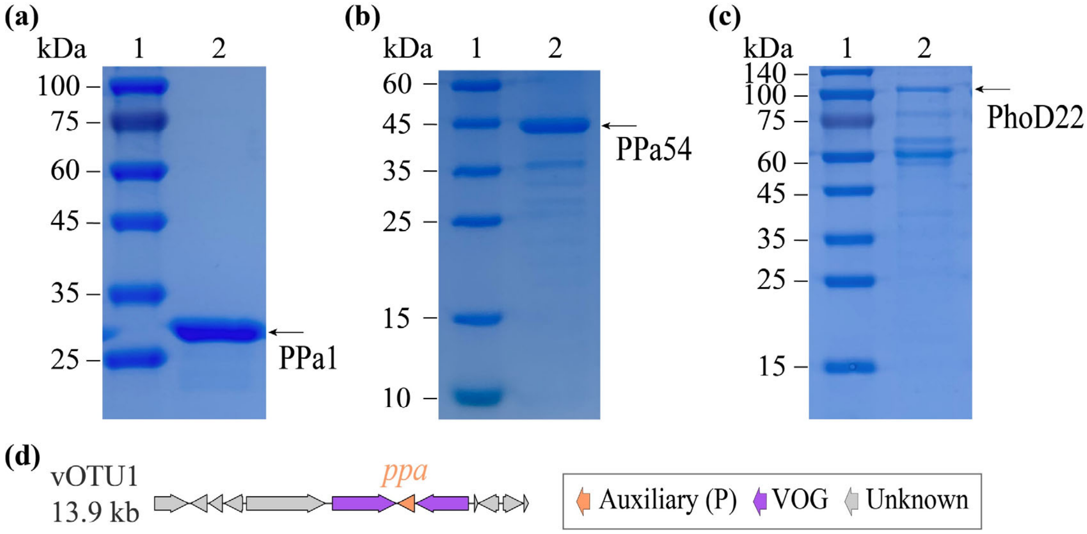

Supplementary Fig. 21 | Products of three representative P-acquisition AMGs expressed in *Escherichia coli*. (a–c) Coomassie-stained SDS polyacrylamide gel electrophoresis of the products of phage *ppa*1 **(a)**, *ppa*54 **(b)**, and *phoD*22 **(c)** expressed in *E. coli*. Lane 1, markers; lanes 2, products (indicated by straight arrows). **(d)** Genome organization diagram of the vOTU1 that encoded *ppa*1. P, phosphorus. AMGs, auxiliary metabolic genes. Predicted open reading frames are colored according to VIBRANT and DRAM-v annotation functions. vOTU, viral operational taxonomic unit. VOG, virus orthologous groups.

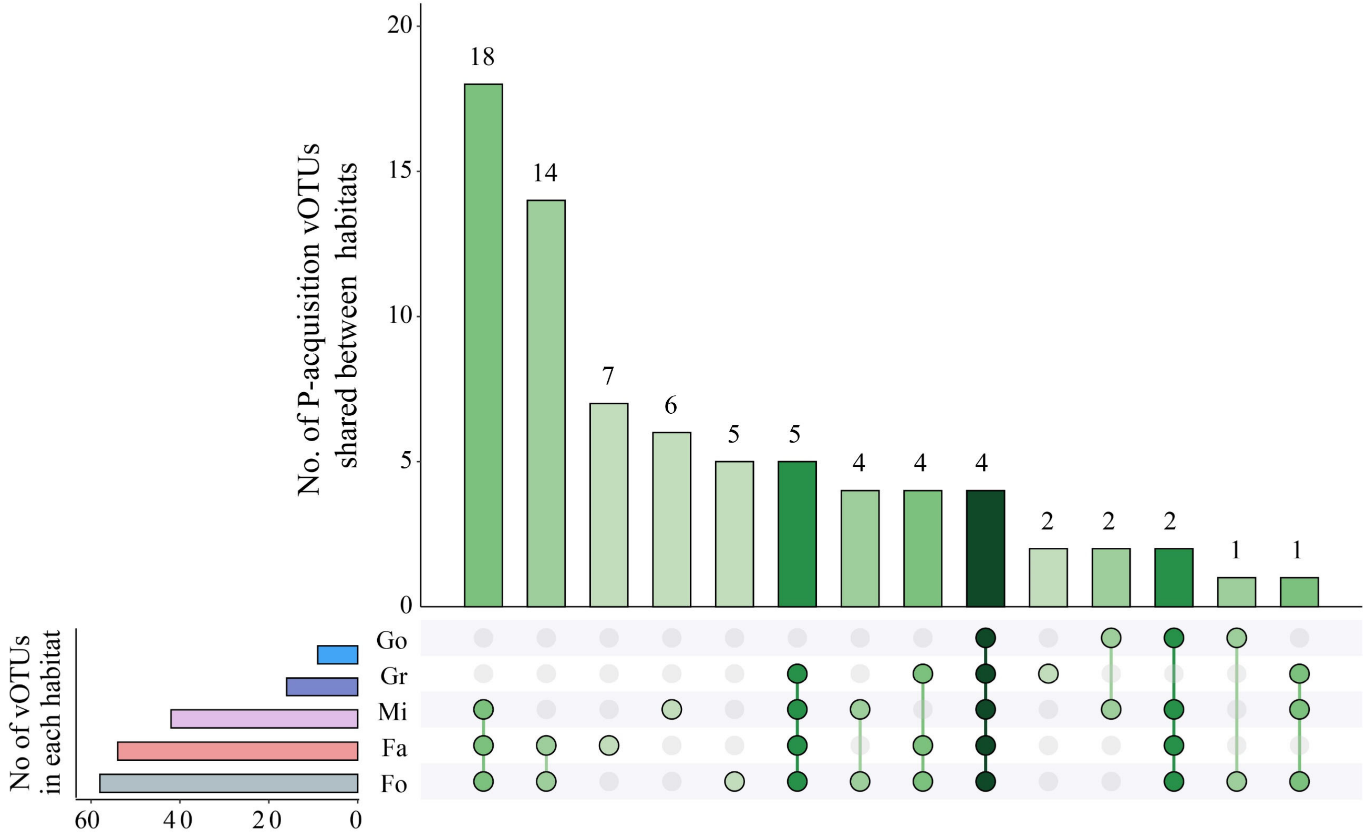

Supplementary Fig. 22 | Ubiquity and uniqueness of soil P-acquisition vOTUs. Shared and unique P-acquisition vOTUs of the five different habitat types. Fa, farmland; Fo, forest; Mi, mine wasteland; Gr, grassland; Go, Gobi desert.

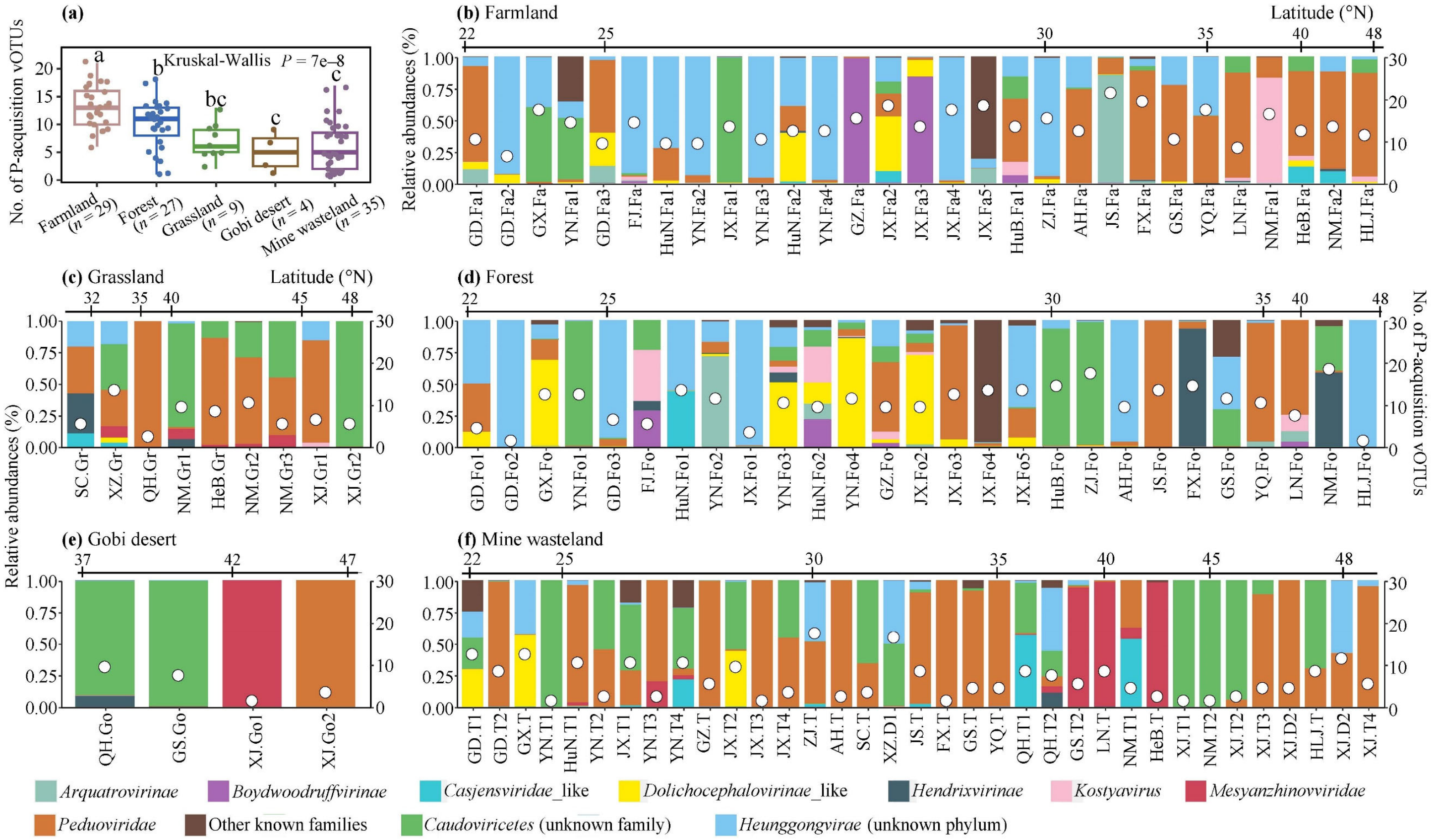

Supplementary Fig. 23 | The numbers and community compositions of soil P-acquisition vOTUs in individual sampling sites. (a) The average numbers of P-acquisition vOTUs detected in five habitat types. Horizontal lines represent the medians, whereas the boxes represent the interquartile ranges of the first and third quartiles. The vertical lines represent the maximal and minimal values. Different letters on the top of the

bars indicate significant differences between habitat types assessed with two-sided Wilcox test, and *P* value indicates the global significance assessed with the Kruskal-Wallis test. **(b-f)** The community compositions of P-acquisition vOTUs in individual sampling sites. The relative abundances of various phage families are shown in the bar charts (see scale values on the X-axis of the left-hand side of each panel) and the numbers of P-acquisition vOTUs detected are illustrated with white circles (see scale values on the X-axis of the right-hand side of each panel). Sampling sites are first grouped as per their habitat types [Farmland **(b)**, Forest **(c)**, Grassland **(d)**, Gobi desert **(e)**, and Mine wasteland **(f)**], and then those within the same habitat type are arranged according to their latitudes (from south to north). Relative abundances of each P-acquisition vOTU were provided in Supplementary Data 14.

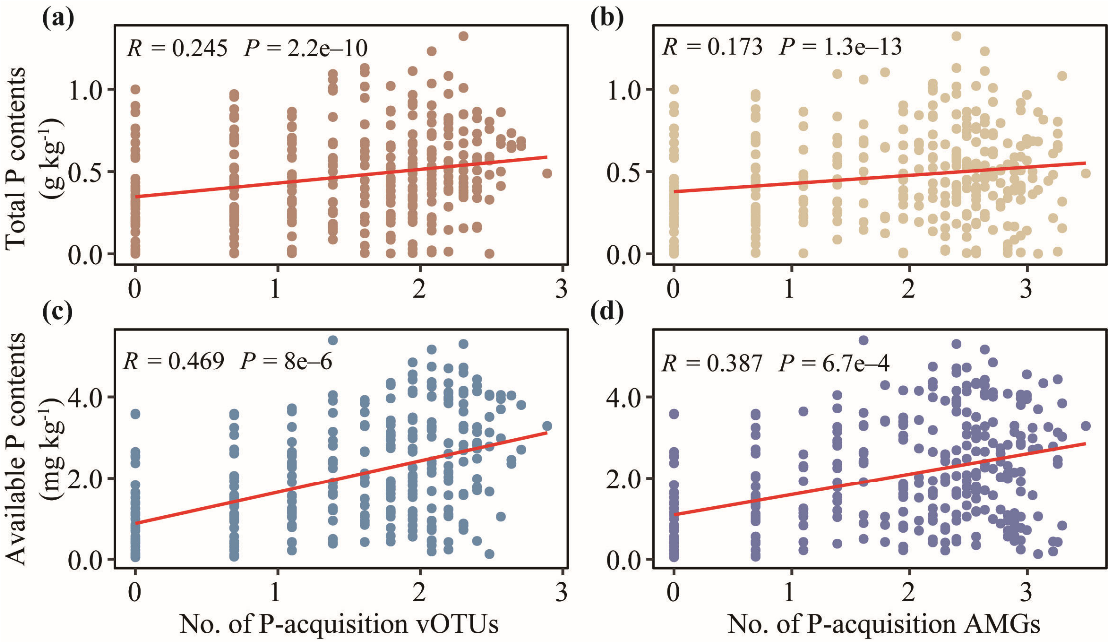

Supplementary Fig. 24 | Correlations between soil phosphorous (P) contents and the numbers of P-acquisition vOTUs (a and c panels) or AMGs (b and d panels) detected in our soil metagenomes. Each dot represents on the panels one soil sample. The solid red lines represent the linear regressions with statistically significant Pearson coefficients. All data were transformed with a log-transformation (ln(data + 1)).

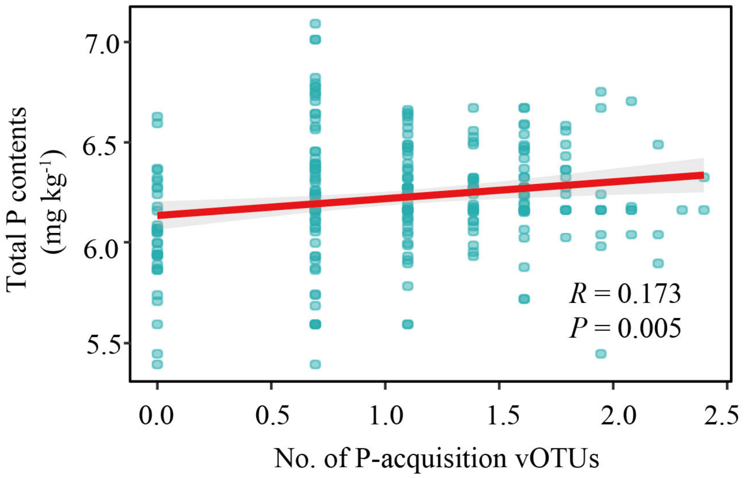

Supplementary Fig. 25 | Correlation between total soil phosphorous contents and the numbers of P-acquisition vOTUs detected in published global topsoil metagenomes. Each dot represents one soil sample from a published global topsoil metagenome dataset[13]. The solid red line represents the linear regression with a statistically significant Pearson coefficient. All data were transformed with a log-transformation (ln(data + 1)). The total P contents of individual soil samples were obtained from a previous study that presented a global map of soil total P content[14].

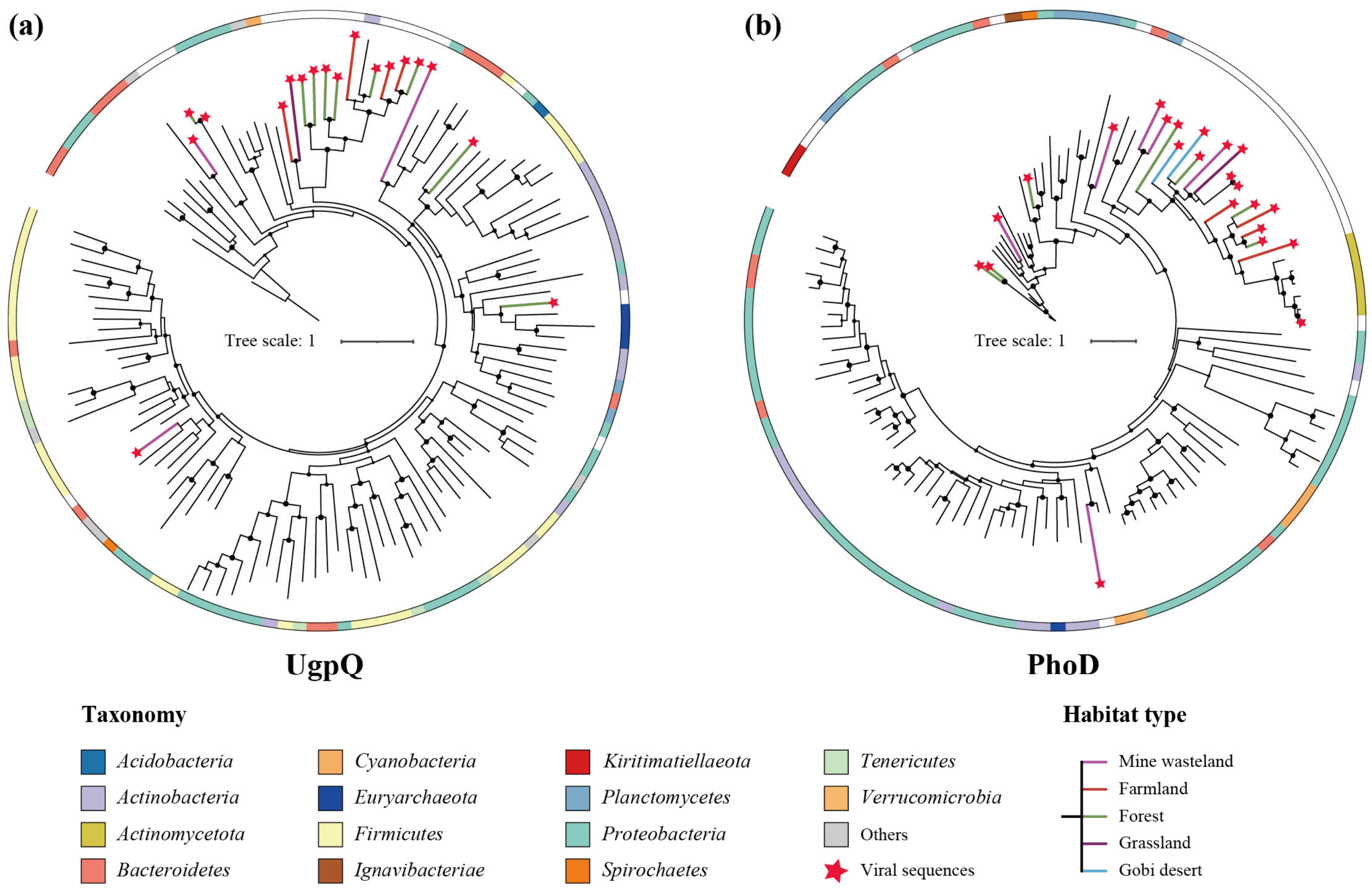

Supplementary Fig. 26 | Phylogenetic trees of UgpQ (a) and PhoD (b). Bootstrap values > 75% are labeled on the nodes. Proteins encoded by the P-acquisition vOTUs identified in this study are labeled with stars and their habitat origins indicated by colored branches. Branches colored in

black represent the phylogenetic placement of the reference prokaryotic sequences. Outside rings denote the taxonomic affiliations of the prokaryotic proteins.

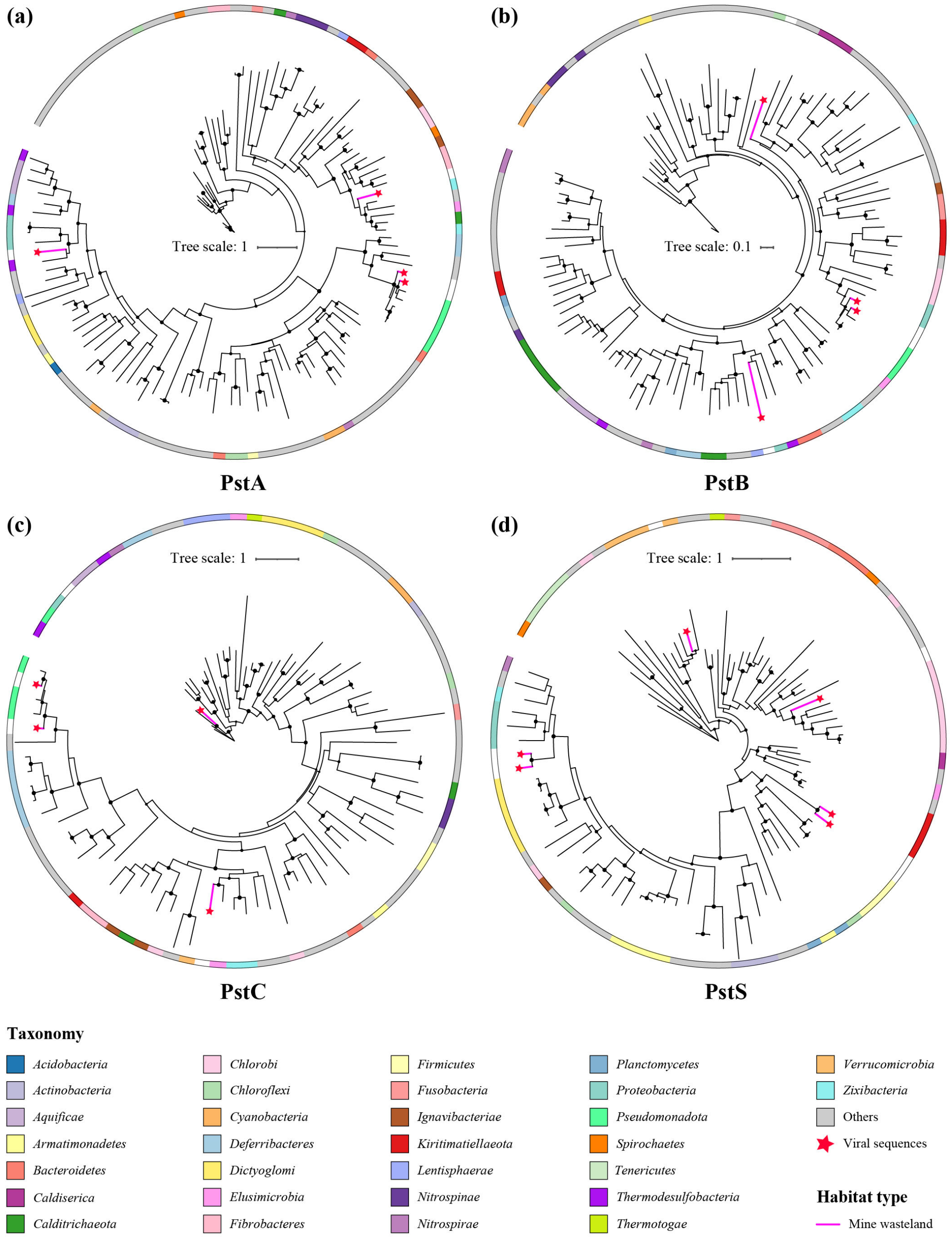

Supplementary Fig. 27 | Phylogenetic trees of PstA (a), PstB (b), PstC (c), and PstS (d). Bootstrap values > 75% are labeled on the nodes. Proteins encoded by the P-acquisition vOTUs identified in this study are labeled with stars and their habitat origins indicated by colored branches. Branches colored in black represent the phylogenetic placement of the reference prokaryotic sequences. Outside rings denote the taxonomic affiliations of the prokaryotic proteins.

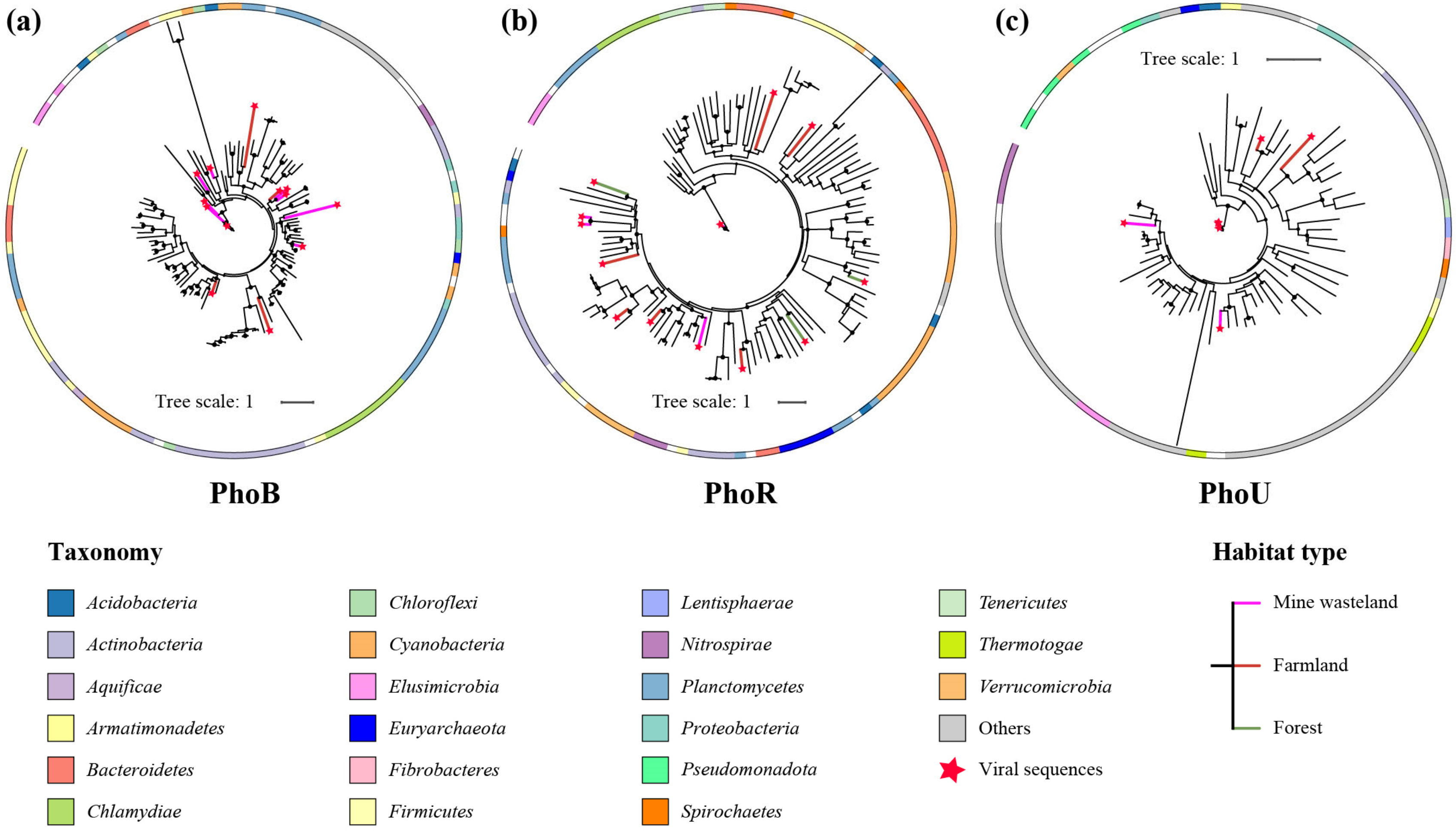

Supplementary Fig. 28 | Phylogenetic trees of PhoB (a), PhoR (b), and PhoU (c). Bootstrap values > 75% are labeled on the nodes. Proteins

encoded by the P-acquisition vOTUs identified in this study are labeled with stars and their habitat origins indicated by colored branches. Branches colored in black represent the phylogenetic placement of the reference prokaryotic sequences. Outside rings denote the taxonomic affiliations of the prokaryotic proteins.

Supplementary references

1. Tommi, K., Juho, K. & Adrian, G. Inorganic pyrophosphatases: one substrate, three mechanisms. *FEBS Lett.* **587**, 1863–1869 (2013).

2. Jia, R. L., Zhao, Y. M. & Hattori, M. Crystal structure of the catalytic ATP-binding domain of the PhoR sensor histidine kinase. *Proteins* **91**, 999–1004 (2023).

3. Rodriguez, F. et al. Crystal structure of the *Bacillus subtilis* phosphodiesterase PhoD reveals an iron and calcium-containing active site. *J. Biol. Chem.* **289**, 30889–30899 (2014).

4. Brautigam, C. A., Ouyang, Z. M., Deka, R. K. & Norgard, M. V. Sequence, biophysical, and structural analyses of the PstS lipoprotein (BB0215) from *Borrelia burgdorferi* reveal a likely binding component of an ABC-type phosphate transporter. *Protein Sci.* **23**, 200–212 (2014).

5. Bisson, C. et al. The molecular basis of phosphite and hypophosphite recognition by ABC-transporters. *Nat. Commun.* **8**, 1746 (2017).

6. Beaudoin, G. A. W., Li, Q., Bruner, S. D. & Hanson, A. D. An unusual diphosphatase from the PhnP family cleaves reactive FAD photoproducts. *Biochem. J.* **475**, 261–272 (2018).

7. Kelley, L. A., Mezulis, S., Yates, C. M., Wass, M. N. & Sternberg, M. J. E. The Phyre2 web portal for protein modeling, prediction and analysis. *Nat. Protoc.* **10**, 845–858 (2015).

8. Okajima, T. et al. Response regulator YycF essential for bacterial growth: X-ray crystal structure of the DNA-binding domain and its PhoB-like DNA recognition motif. *FEBS Lett.* **582**, 3434–3438 (2008).

9. Lee, S. J., Park, Y. S., Kim, S.-J., Lee, B.-J. & Suh, S. W. Crystal structure of PhoU

from *Pseudomonas aeruginosa*, a negative regulator of the Pho regulon. *J. Struct. Biol.* **188**, 22–29 (2014).

10. Tsai, J.-Y. et al. Structure of the sodium-dependent phosphate transporter reveals insights into human solute carrier SLC20. *Sci. Adv.* **6**, eabb4024 (2020).

11. Rangarajan, E. S. et al. The structure of the exopolyphosphatase (PPX) from *Escherichia coli* O157:H7 suggests a binding mode for long polyphosphate chains. *J. Mol. Biol.* **359**, 1249–1260 (2006).

12. Shi, L., Liu, J.-F., An, X.-M. & Liang, D.-C. Crystal structure of glycerophosphodiester phosphodiesterase (GDPD) from *Thermoanaerobacter tengcongensis*, a metal ion-dependent enzyme: insight into the catalytic mechanism. *Proteins* **72**, 280–288 (2008).

13. Bahram, M. et al. Structure and function of the global topsoil microbiome. *Nature* **560**, 233–237 (2018).

14. He, X. J. et al. Global patterns and drivers of soil total phosphorus concentration. *Earth Syst. Sci. Data* **13**, 5831–5846 (2021).
