# Supplementary material for: Hidden diversity and potential ecological function of phosphorus acquisition genes in widespread terrestrial bacteriophages
Source: Nat Commun. 2024 Apr 2;15:2827. doi: 10.1038/s41467-024-47214-7 (PMC10987575; doi:10.1038/s41467-024-47214-7)
Supplement: Supplementary file 3 — Description of Additional Supplementary Files [file 41467_2024_47214_MOESM3_ESM.pdf]

Description of Additional Supplementary Files

Supplementary Data Legends

File Name: Supplementary Data 1

Description: Detailed information of the sampling sites of this study and selected environmental factors of these sites.

File Name: Supplementary Data 2

Description: Detailed information of the genes related to the four processes of microbial P-acquisition investigated in this study.

File Name: Supplementary Data 3

Description: HMM databases and the corresponsive cutoff scores used for validation of P-acquisition genes.

File Name: Supplementary Data 4

Description: Detailed information of the 75 P-acquisition vOTUs identified in this study.

File Name: Supplementary Data 5

Description: Genomic arrangements of the 75 P-acquisition vOTUs identified in this study (annotated by VIBRANT).

File Name: Supplementary Data 6

Description: DRAM-v annotations of the 75 P-acquisition vOTUs identified in this study.

File Name: Supplementary Data 7

Description: Information of the AMGs encoded by the 75 P-acquisition vOTUs identified in this study.

File Name: Supplementary Data 8

Description: Taxonomic affiliations of the 75 P-acquisition vOTUs identified in this study.

File Name: Supplementary Data 9

Description: Summary of the P-acquisition AMGs reported previously.

File Name: Supplementary Data 10

Description: Information of the P-acquisition AMGs encoded by the 106 published viral genomes.

File Name: Supplementary Data 11

Description: DRAM-v annotations of the three public viral genomes displayed in Fig. 2c.

File Name: Supplementary Data 12

Description: Information of computational protein models illustrated in Fig. 3 and Supplementary Fig. 19.

File Name: Supplementary Data 13

Description: Phage P-acquisition AMG pairs and dN/dS calculations. The table header corresponds to the output generated from dnds_from_drep.py (as per Olm et al, 2020).

File Name: Supplementary Data 14

Description: Relative abundances of the 75 P-acquisition vOTUs identified in this study.

File Name: Supplementary Data 15

Description: Information of the 27 P-acquisition vOTUs mapped in the global topsoil metagenomes.

File Name: Supplementary Data 16

Description: The P-acquisition AMGs and their corresponsive vOTUs mapped in the global topsoil metagenomes.

File Name: Supplementary Data 17

Description: Information of P-acquisition vOTUs-host linkages illustrated in Fig. 7a.

File Name: Supplementary Data 18

Description: Information of the 32 public metatranscriptomic datasets analyzed in this study.

File Name: Supplementary Data 19

Description: The P-acquisition AMGs detected in the public metatranscriptomic datasets.
