# Supplementary material for: Hidden diversity and potential ecological function of phosphorus acquisition genes in widespread terrestrial bacteriophages
Source: Nat Commun. 2024 Apr 2;15:2827. doi: 10.1038/s41467-024-47214-7 (PMC10987575; doi:10.1038/s41467-024-47214-7)
Supplement: Supplementary file 5 — Reporting Summary [file 41467_2024_47214_MOESM5_ESM.pdf]

nature portfolio

Corresponding author(s): Jin-tian Li

Last updated by author(s): Mar 10, 2024

Reporting Summary

Nature Portfolio wishes to improve the reproducibility of the work that we publish. This form provides structure for consistency and transparency in reporting. For further information on Nature Portfolio policies, see our Editorial Policies and the Editorial Policy Checklist.

Statistics

For all statistical analyses, confirm that the following items are present in the figure legend, table legend, main text, or Methods section.

n/a	Confirmed	
☐	☒	The exact sample size (*n*) for each experimental group/condition, given as a discrete number and unit of measurement
☐	☒	A statement on whether measurements were taken from distinct samples or whether the same sample was measured repeatedly
☐	☒	The statistical test(s) used AND whether they are one- or two-sided<br>*Only common tests should be described solely by name; describe more complex techniques in the Methods section.*
☒	☐	A description of all covariates tested
☐	☒	A description of any assumptions or corrections, such as tests of normality and adjustment for multiple comparisons
☐	☒	A full description of the statistical parameters including central tendency (e.g. means) or other basic estimates (e.g. regression coefficient) AND variation (e.g. standard deviation) or associated estimates of uncertainty (e.g. confidence intervals)
☐	☒	For null hypothesis testing, the test statistic (e.g. *F*, *t*, *r*) with confidence intervals, effect sizes, degrees of freedom and *P* value noted<br>*Give P values as exact values whenever suitable.*
☒	☐	For Bayesian analysis, information on the choice of priors and Markov chain Monte Carlo settings
☐	☒	For hierarchical and complex designs, identification of the appropriate level for tests and full reporting of outcomes
☐	☒	Estimates of effect sizes (e.g. Cohen's *d*, Pearson's *r*), indicating how they were calculated

Our web collection on statistics for biologists contains articles on many of the points above.

Software and code

Policy information about availability of computer code

Data collection	No software was used for data collection.
Data analysis	Open source tools:<br>1. Assembling of short reads: MEGAHIT (version 1.2.9)<br>2. ORFs predicting: MetaProdigal (version 2.6.3)<br>3. Identification of viral sequences: VirSorter2 (version 2.2.3) and VIBRANT (version 1.2.1)<br>4. Quality-checked of viral sequences: CheckV (version 0.9.0)<br>5. Clustering of viral sequences: dRep (version 3.3.0)<br>6. Gene function annotation: Diamond (version 0.9.24.125) and HMMER 3.3.2<br>7. AMG validation: DRAM (version 1.2.0)<br>8. Clustering of genes: CD-HIT (version 4.8.1)<br>9. Taxonomic assignment of vOTU: geNomad (version 1.7.0) and PhaGCN (version 2.0)<br>10. Sequence alignment: MAFFT (version 7.490) and ClustalW (https://www.genome.jp/tools-bin/clustalw)<br>11. Construction of phylogenetic trees: IQ-TREE (version 1.6.12)<br>12. Editing of phylogenetic tree topology: the Interactive Tree of Life online interface (https://itol.embl.de/)<br>13. Protein structure model: PHYRE2 (http://www.sbg.bio.ic.ac.uk/phyre2/)<br>14. Reads mapping: Bowtie2 (version 2.3.4.1)<br>15. Quality-control of metatranscriptomic reads: fastp (version 0.23.2) and SortMeRNA (version 4.3.6)<br>16. Genome binning, assessment, and phylogeny: metaBAT2 (version 2.12.1), CheckM (version 1.2.0), and GTDB-Tk (version 2.1.1)<br>17. Host prediction: iPHoP (version 1.3.3)

18. Statistical analysis: R software 4.1.0
19. In-house code: https://doi.org/10.5281/zenodo.10746127

For manuscripts utilizing custom algorithms or software that are central to the research but not yet described in published literature, software must be made available to editors and reviewers. We strongly encourage code deposition in a community repository (e.g. GitHub). See the Nature Portfolio guidelines for submitting code & software for further information.

Data

Policy information about availability of data

All manuscripts must include a data availability statement. This statement should provide the following information, where applicable:

- Accession codes, unique identifiers, or web links for publicly available datasets
- A description of any restrictions on data availability
- For clinical datasets or third party data, please ensure that the statement adheres to our policy

Metagenomic sequencing data have been deposited in NCBI BioProject database under accession code PRJNA1085405 [https://www.ncbi.nlm.nih.gov/bioproject/PRJNA1085405]. The vOTU sequences have been deposited in the European Bioinformatics InstituteENA Sequence Read Archive database under the accession numbers of PRJEB60228 [https://www.ebi.ac.uk/ena/browser/view/PRJEB60228]. Previous published 288 global topsoil metagenomes are available in ENA Sequence Read Archive database under the accession number of PRJEB24121 [https://www.ebi.ac.uk/ena/browser/view/PRJEB24121]. Previous published 32 metatranscriptomes are available in ENA and NCBI Sequence Read Archive databases (PRJNA716119, PRJNA1056670, and PRJEB42658) and the accession number for each sample are listed in Supplementary Data 18. Datasets used and/or analyzed during the study can be found in Supplementary Data 1-19.

Research involving human participants, their data, or biological material

Policy information about studies with human participants or human data. See also policy information about sex, gender (identity/presentation), and sexual orientation and race, ethnicity and racism.

Reporting on sex and gender	Not relevant to our study.
Reporting on race, ethnicity, or other socially relevant groupings	Not relevant to our study.
Population characteristics	Not relevant to our study.
Recruitment	Not relevant to our study.
Ethics oversight	Not relevant to our study.

Note that full information on the approval of the study protocol must also be provided in the manuscript.

Field-specific reporting

Please select the one below that is the best fit for your research. If you are not sure, read the appropriate sections before making your selection.

☐ Life sciences ☐ Behavioural & social sciences ☒ Ecological, evolutionary & environmental sciences

For a reference copy of the document with all sections, see nature.com/documents/nr-reporting-summary-flat.pdf

Ecological, evolutionary & environmental sciences study design

All studies must disclose on these points even when the disclosure is negative.

Study description	In this study, we collected 333 soil samples from five distinctive terrestrial habitat types across China, including 29 farmland, 27 forest, 4 Gobi desert, 3 grassland and 42 mine wasteland ecosystems. We identified 75 viral operational taxonomic units (vOTUs) that encoded 105 P-acquisition AMGs from 333 soil metagenomes. Our results shed new light on the ecology of P-acquisition AMGs and reinforce the necessity of incorporating viral contributions into biogeochemical P cycling.
Research sample	We chose three habitat types (i.e., farmland, forest, and grassland) that were slightly deficient in P, and two habitat types (i.e., Gobi desert and mine wasteland) that were extremely deficient in P. These soil samples were collected from as many as 22 provinces across China, to make the datasets more representative.
Sampling strategy	At each study site, two to four types of terrestrial habitat were chosen for sampling when they were simultaneously distributed within an area of approximately 25 km2. At each ecosystem, we collected three soil samples at a depth of 0–20 cm according to the method described previously (Lu et al., 2022).
Data collection	Soil samples were collected by SWF and JLL. Soil DNA was extracted by YQG, SYL, YYZ, using the FastDNA Spin kit (MP Biomedicals, Santa Ana, CA, USA) following the manufacturer's protocol. DNA quality was assessed by SYL using the NanoDrop 2000 spectrophotometer (Thermo Scientific, USA). The metagenome sequencing library was prepared by SWF and shotgun-sequenced on Illumina HiSeq 2500 platform with PE150 mode (Illumina, USA). Sequencing reads were processed by J-LL for quality control, scaffold

	assembly, viral sequence identification and ecological analyses.<br>Bioavailable and total soil P contents were determined by JLL according to sodium bicarbonate (Olsen) method and molybdate blue colorimetric method (Lu et al, 2022).
Timing and spatial scale	Soil samples were collected between July and August 2018 on a country scale.
Data exclusions	No data was excluded.
Reproducibility	Enzyme activities were successfully confirmed with three replicates using distinct samples.
Randomization	It's not relevant to our study because we conducted random sampling across China and the samples were not allocated into groups. The number of replicates equals the number of sample in our case.
Blinding	During data analysis, blinding was conducted by only taking the sample ID into account and the sampling location was not involved.

Did the study involve field work? ☒ Yes ☐ No

Field work, collection and transport

Field conditions	The climatic conditions of the sampling sites varied considerably, with mean annual precipitation ranging from 83 to 1,730 mm.
Location	The sampling sites covered latitude from 23.9°N to 47.7°N, and longitude from 88.1°E to 129°E.
Access & import/export	Import/export is not relevant to our study.
Disturbance	Only topsoils (i.e., 0-20 cm in depth) were collected, and each sample was only 10 g. No disturbances are caused by this study.

Reporting for specific materials, systems and methods

We require information from authors about some types of materials, experimental systems and methods used in many studies. Here, indicate whether each material, system or method listed is relevant to your study. If you are not sure if a list item applies to your research, read the appropriate section before selecting a response.

Materials & experimental systems

n/a	Involved in the study	
☒	☐	Antibodies
☒	☐	Eukaryotic cell lines
☒	☐	Palaeontology and archaeology
☒	☐	Animals and other organisms
☒	☐	Clinical data
☒	☐	Dual use research of concern
☒	☐	Plants

Methods

n/a	Involved in the study	
☒	☐	ChIP-seq
☒	☐	Flow cytometry
☒	☐	MRI-based neuroimaging

Plants

Seed stocks	Not relevant to our study.
Novel plant genotypes	Not relevant to our study.
Authentication	Not relevant to our study.
